# Unifying sequence-structure coding for advanced protein engineering *via* a multimodal diffusion transformer†

**DOI:** 10.1039/d5sc02055g

**Published:** 2025-05-15

**Authors:** Xiaohan Lin, Zhenyu Chen, Yanheng Li, Zicheng Ma, Chuanliu Fan, Ziqiang Cao, Shihao Feng, Jun Zhang, Yi Qin Gao

**Affiliations:** a Beijing National Laboratory for Molecular Sciences, College of Chemistry and Molecular Engineering, Peking University Beijing 100871 China gaoyq@pku.edu.cn; b Changping Laboratory Beijing 102200 China fengsh@cpl.ac.cn jzhang@cpl.ac.cn; c Academy for Advanced Interdisciplinary Studies, Peking University Beijing 100871 China; d Institute of Artificial Intelligence, Soochow University Suzhou 215006 China

## Abstract

Modern protein engineering demands integrated sequence–structure representations to tackle key challenges in designing, modifying, and evolving proteins for specific functions. While sequence-based methods are promising for generating novel proteins, incorporating structure-oriented information improves the success rate and helps target corresponding functions. Therefore, rather than relying solely on sequence or structure-based approaches, a consensus strategy is essential. Here, we introduce ProTokens, machine-learned “amino acids” derived from structural databases *via* self-supervised learning, providing a compact yet information-rich representation that bridges sequence and structure modalities. Instead of treating sequences and structures separately, we build PT-DiT, a multimodal diffusion transformer-based model that integrates both into a unified representation, enabling protein engineering in a joint sequence–structure space, streamlining the design process and facilitating the efficient encoding of 3D folds, contextual protein design, sampling of metastable states, and directed evolution for diverse objectives. Therefore, as a unified solution for *in silico* protein engineering, PT-DiT leverages sequence and structure insights to realize functional protein design.

## Abstract 2

This study introduces ProTokens, machine-learned “amino acids” for protein structure representation, and PT-DiT, a multimodal diffusion transformer for unified sequence–structure modeling, design, and evolution.

## Graphical abstract

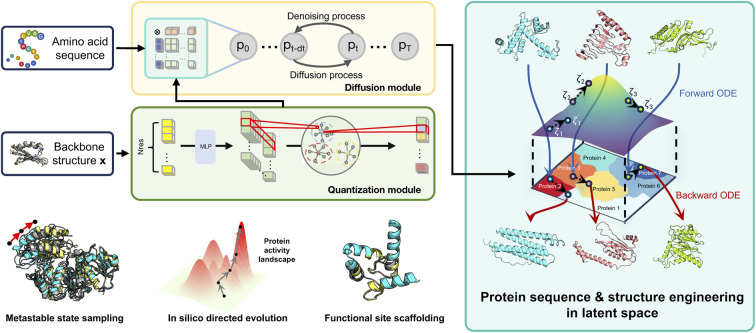

## Introduction

Computation-aided protein engineering, encompassing the design, modification, and evolution of proteins for specific functions, is invaluable in scientific research and medical development.^1–4^ Successfully designing functional proteins can lead to the creation of beneficial therapeutic or industrial biomolecules, such as antibodies and enzymes.^5–10^ However, there is ongoing debate about whether sequence-based or structure-based approaches are more effective for *in silico* protein engineering.^11^ Structure-oriented approaches are often considered more “informative”, as the function of proteins is largely determined by their three-dimensional (3D) structures. Yet, accurately describing protein structures remains a significant challenge due to the redundancy and irregularity of atomic coordinates, which are difficult to design and edit because of symmetry and physical constraints. In contrast, sequences—combinations of amino acids—offer compact and machine-friendly representations that are compatible with most computational models, including large language models (LLMs). Although models like ESM^12,13^ and ProteinBERT^14^ have achieved remarkable success in sequence-based protein modeling, the connection between functions and sequences often remains ambiguous, as biologically, proteins must be correctly folded to perform their functions effectively. For example, sequence-based representations fail to distinguish different functional conformations of proteins,^15,16^ which are crucial for their proper functionality.

Although structure prediction models like AlphaFold^17–19^ and RoseTTAFold^20^ have demonstrated their ability to map sequences to structures, the quality of their folding largely relies on homologous sequences and structural templates. On the other hand, inverse folding models such as ProteinMPNN^6^ can design sequences that stabilize and accommodate given backbone structures, but their generalizability to rare and novel folds remains to be tested. Consequently, the modality difference of protein representations causes significant divergence in the research paradigms of proteins, particularly in the realm of protein design. Thus, bridging the gap between sequence and structure modalities remains a significant challenge and limits applications such as the generation of diverse and novel *de novo* proteins.^13,21^

Aiming to provide a unified perspective on protein sequences and structures, we developed here ProTokens. Conceptually, ProTokens represent a novel set of “amino acids” learned and extracted from protein structure databases through self-supervised learning. As machine-learned amino acids, ProTokens offer compact informative representations of protein structures. They possess the technical advantages of sequence representations, being compact for storage and convenient for use as input/output in computational models. Furthermore, through a sophisticatedly designed training strategy, ProTokens are as informative as foldable 3D protein structures. Protein structures are efficiently compressed into sequences of ProTokens, which can then be accurately and reliably “folded” back into their corresponding structures.

By harnessing the computational convenience of sequence-like representations and the functional relevance of structure-awareness, ProTokens naturally bridge the gap between sequence-based and structure-based methodologies in protein engineering. By combining natural amino acids with ProTokens, we train a diffusion transformer^22^-based model PT-DiT, to model the joint probability of protein sequences and structures. Leveraging this generative objective, we found PT-DiT to be a versatile tool for protein engineering at both the sequence and structure levels, enabling design with residue-wise protein contexts for tasks such as contextual inverse folding and functional site scaffolding. Similar to protein language models, generative pre-training of PT-DiT yields a powerful latent representation that jointly and faithfully embeds sequences and structures. Utilizing zero-shot or few-shot learning on this representation, PT-DiT's capabilities extend to sampling metastable states in protein dynamics, rediscovering naturally occurring or *de novo* “evolutionary” intermediates in remote homologs, and directing the evolution of proteins towards specific objectives.

## Results

### Generative, differentiable ProToken and PT-DiT architecture for protein sequence–structure co-engineering

**Fig. 1:**
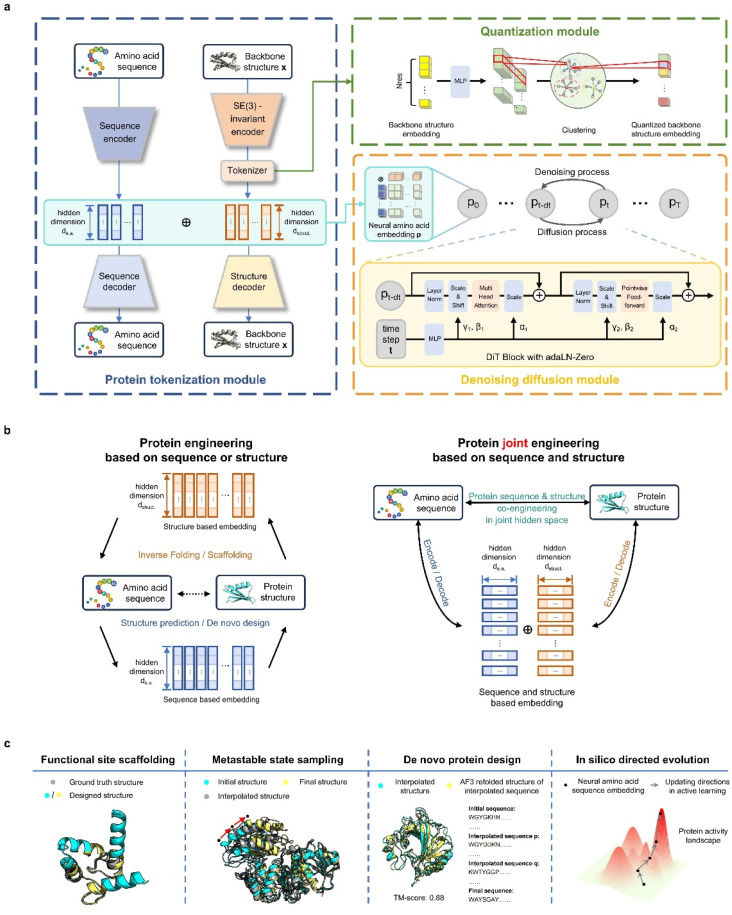
Schematic overview of protein engineering with ProTokens. (a) PT-DiT workflow. The protein tokenization module employs a twin-tower embedding model, which pre-trains embeddings in both sequence and structure modalities. The quantization module compresses the structure embeddings. The denoising diffusion module, the core of PT-DiT, models the joint probability of sequences and structures, enabling the co-engineering of protein sequences and structures. (b) Comparison of sequence and structure integration in protein engineering approaches. Traditional methods treat sequences and structures as separate entities, connecting them through additional folding and inverse folding models (left panel). In contrast, our approach uses neural amino acids (ProTokens) to extend the vocabulary of naturally occurring amino acids, offering a more informative, joint representation of sequences and structures. This allows the projection of both into a unified space for seamless integration. (c) ProTokens and PT-DiT as versatile tools for protein engineering, enabling tasks such as functional site scaffolding, multi-conformational structure sampling, *de novo* protein design, and *in silico* directed evolution.

Our contributions are twofold: (1) we propose and pretrain a structure-aware amino-acid-like representation, ProToken, that compresses the foldable protein structures to 512 tokens with high fidelity (reconstruction TM-score > 0.90) (Fig. 1a); and (2) we unify the representations of protein sequences and structures, enabling integrated protein engineering in a shared sequence–structure space through a diffusion transformer model PT-DiT (Fig. 1b and c).

ProTokens are learned *via* a vector-quantized autoencoding^23^ framework trained on a backbone reconstruction task using 3D structural data from metastable conformations of foldable proteins (Fig. 1a). Each residue in a given structure is mapped to a discrete ProToken selected from a constrained codebook, capturing residue-level structural features. Notably, the input to the encoder consists solely of backbone atom coordinates (N, CA, C, O), ensuring that the ProToken representation encodes sequence-agnostic geometric information. Unlike naturally occurring amino acids, ProTokens can be readily decoded into atomistic coordinates. During training, ProTokens that capture different local and global structural features remain distinguishable, with the training objective driving ProTokens representing similar conformations closer together in latent space. This strategy yields a more nuanced representation of conformational ensembles. Once trained, ProTokens enable efficient compression, storage, alignment, and comparison of protein structures.

PT-DiT aims to engineer protein sequences, structures, or both from a unified generative perspective, regardless of whether sequence or structural information is initially available (Fig. 1b). Built upon a pretrained twin-tower embedding model, we use ProTokens (structure-aware embeddings) and natural amino acid embeddings as dual-channel representations of proteins. The structure embeddings are derived from protein backbone structures *via* the ProToken encoder, while the sequence embeddings are computed using a PCA-compressed of AlphaFold2 (ref. 18) or ESMFold^12^ embeddings. The ProToken embeddings and sequence embeddings are concatenated into a joint representation for each residue, which serves as the input to train a diffusion-based generative model, PT-DiT (see Methods). Drawing inspiration from image diffusion methods such as RePaint^24^ and probability flow ordinary differential equations,^25^ PT-DiT models the joint distribution of protein sequences and structures, enabling a wide range of tasks including *de novo* design, scaffolding, metastable state sampling, and directed evolution. In contrast to traditional approaches that treat sequences and structures as separate modalities^19,26^ linked only by computational folding or inverse folding, PT-DiT integrates both into a unified representation. This integration enables protein engineering in a joint sequence–structure space, streamlining the design process and facilitating the derivation of specialized functions.

We introduce a probabilistic framework to clarify the methodology underlying structure-informed representations (see the ESI†), establishing the basis for the ProToken and PT-DiT algorithms. To ensure uniqueness, compactness, and sufficiency of the ProToken code, we incorporate alignment and uniformity loss functions^27^ (Fig. S1†). A test set was curated for the reconstruction task, enabling evaluation of our protein tokenization module's performance and generalizability using structures from CASP14^28^ and the RCSB database,^29^ and AFDB^30^ dark clusters identified by Foldseek.^26^ TM-score^31^ and LDDT^32^ were computed between the reconstructed and original structures (see Methods) to benchmark performance. We subsequently demonstrate PT-DiT's feasibility for traditional tasks such as inverse folding and contextual backbone design. We then comprehensively benchmark PT-DiT's performance across three main applications: (a) metastable conformation sampling, validated by molecular dynamics simulations and experimental data; (b) *de novo* protein sequence design *via* latent space interpolation between remote homologs; and (c) directed evolution using a state-of-the-art active learning pipeline, benchmarked with EVOLVEpro.^33^

### ProTokens are concise representations of metastable protein structures

**Fig. 2:**
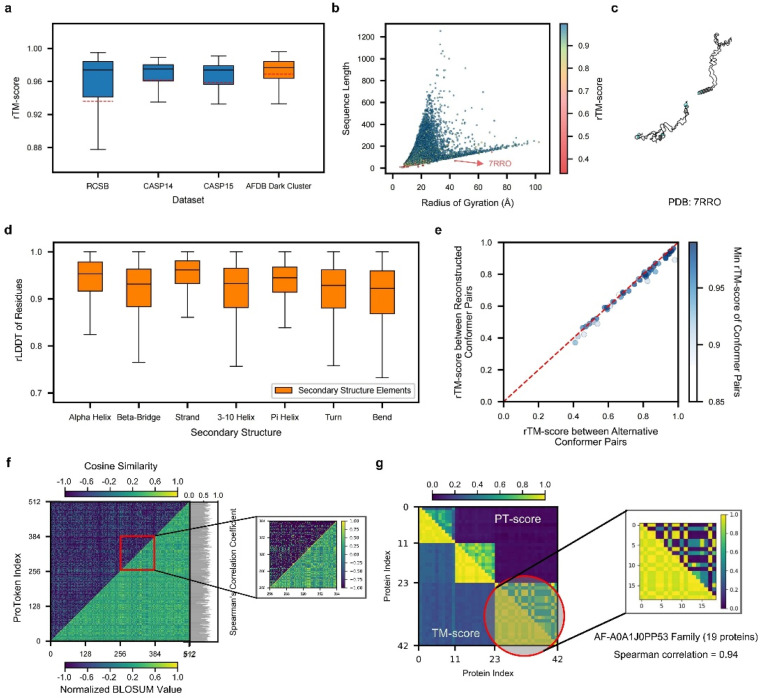
Performance of ProTokens on reconstruction test datasets. (a) The rTM-score distribution of ProToken reconstruction across 513, 87, 45, and 33 842 samples for RCSB, CASP14, CASP15 and AFDB dark cluster datasets, respectively. (b) The rTM-score distribution of 34 487 protein samples in four reconstruction test datasets with different sequence lengths and radii of gyration. The red arrow indicates the example protein (PDB ID 7RRO) which ProToken fails to reconstruct. The color denotes the rTM-score of each sample. (c) The reconstructed protein structure of 7RRO (cyan) and ground truth structure (gray). (d) The rLDDT distribution of all residues in four reconstruction test datasets across seven types of secondary structures. (e) rTM-score of ProToken reconstruction across 50 conformation pairs from PDBFlex. The color denotes the minimum rTM-score of each reconstruction pair, which intends to show the worst ProToken reconstruction performance towards the conformation pairs. (f) The comparison between the BLOSUM-like matrix of ProTokens (low right) and similarity matrix of ProTokens (upper left). The color denotes the normalized BLOSUM value and cosine similarity, respectively. The Spearman correlation coefficient of each ProToken is calculated and histogrammed on the left. The ProToken index from 256 to 384 is specially zoomed in for ease of pattern recognition. (g) The PT-score of each pair of the 42 protein set is calculated and compared to the TM-score. The color denotes the value of the PT-score and TM-score. The third cluster is specially zoomed in and Spearman correlation coefficient of the TM-score and PT-score is 0.94 over these 19 protein similarity rankings. The box plots in panels (a) and (c) are defined by the median as the centre line, first and third quartiles as the box edges and 1.5 times the inter-quartile range as the whiskers.

To demonstrate the efficacy of ProTokens in 3D structure representation, particularly for backbone conformations, we use the quality of reconstructed protein backbone structures as the evaluation metric. Notably, ProTokens are the first set of tokens specifically designed to represent and reconstruct protein structures without relying on sequential or evolutionary information. Therefore, to ensure a fair and comprehensive benchmark, we curated four test sets from diverse resources. These sets comprise 513 experimentally resolved structures from the RCSB database and CAMEO^34^ (excluding structures released after the training set cutoff), 87 from CASP14, 44 from CASP15, and 33 842 high-quality rare structures from AFDB (details in the ESI†). The median reconstruction TM-scores (rTM-scores) across these sets are as follows: 0.97 for the RCSB and CAMEO set, 0.98 for CASP14, 0.97 for CASP15, and 0.98 for AFDB (Fig. 2a). For comparison, ESM3 achieves an average rTM-score of 0.91 for proteins in CASP15, as reported in previous studies.^35^

We further validated ProToken's reconstruction performance on disconnected domain assemblies from CASP14 and CASP15, as well as multimers from the AF2Complex^36^ benchmark sets. Notably, multi-domain and multimer folds were not included during training. The median rTM-scores for the CASP14 and CASP15 domain assemblies are 0.96 and 0.926, respectively (Fig. S2D†), and the median rTM-score for multimers is 0.891 (Fig. S2E and F†). These findings indicate that ProTokens can robustly generalize and accurately reconstruct a wide variety of protein structures, including challenging domain assemblies and multimers.

To further evaluate ProTokens' capacity to represent diverse protein conformations, we assessed reconstruction performance across proteins of varying shapes (defined based on the radius of gyration) and sequence lengths in four test sets (Fig. 2b). Reconstruction quality remained consistent across different lengths and shapes (Fig. S2A–C†), except in cases of extreme conformations such as highly extended, near-linear forms (Fig. 2c). We also examined ProTokens' reconstruction performance for seven secondary structure classes. The median reconstruction LDDT (rLDDT) exceeded 0.90 for all classes, with ‘alpha helix’ and ‘strand’ exceeding 0.95 (Fig. 2d). These findings indicate that ProTokens do not preferentially favor or discriminate against particular folds or secondary structure features, whether global or local, making it a more generalizable and reliable tool to treat different protein folds or local environments in downstream tasks.

Next, we assessed ProToken's ability to capture alternative conformations by reconstructing proteins with multiple states from 50 PDBFlex^37^ clusters that span local RMSDs of 2.0 Å to 53 Å. The results indicate a median rTM-score of 0.98 across all conformations, with a minimum of 0.88, demonstrating that the structural differences before and after reconstruction are preserved with high fidelity (Fig. 2e). These results suggest that ProTokens are distance-preserving for protein conformations, and therefore they can distinguish different conformations with high resolution. Although the training set does not contain explicit examples of identical sequences with different metastable structures (details in the ESI†), the ability to distinguish such conformations emerges during training, driven by the structural diversity present in the dataset and the discriminative capacity of the ProToken encoder.

Because distinct conformations of the same protein sequence map to different ProToken sequences, we examined how ProToken representations correspond to protein structures. Specifically, we computed a BLOSUM^38^ (blocks dubstitution matrix) for ProTokens based on residue pairs with similar local environments (details in Methods and the ESI†). We then compared these BLOSUM values with the cosine similarity of each pair of ProToken embeddings (Fig. 2f). An average Spearman's correlation coefficient of 0.62 indicates a close connection between the ProToken representation space and local structural environments.

Inspired by Foldseek^26^ and MMseqs,^39^ we implemented a ProToken-based BLOSUM using the Needleman–Wunsch algorithm^40^ to derive the PT-score. We randomly sampled three dark cluster centers of AFDB. For each center, we gather all the similar structures in the RCSB database using Foldseek, which results in 42 proteins in total. We then computed both TM-scores and ProToken similarity scores (PT-scores) for each structure pair (Fig. 2g). Higher PT-scores correlate with higher TM-scores, indicating that the PT-score not only differentiates similar from dissimilar structures but also captures quantitative differences relative to a reference. Thus, the PT-score serves as a promising metric for structure searching, clustering, and analysis algorithms, particularly in applications such as studying long-range allosteric interactions^41^ and protein pocket identification.^42^

### Generative pretraining of ProTokens on a unified perspective of sequences and structures

ProTokens convert 3D protein structures into discrete tokens in a 1D vector space, allowing deep learning frameworks to more readily model the joint probabilities of sequences and structures. Building on this, we trained a diffusion transformer, PT-DiT, using a generative pre-training objective that integrates ProToken embeddings with sequence embeddings derived from ESM-2 (ref. 12) and AlphaFold2 (ref. 18) (see Methods), respectively. As a result, PT-DiT functions not only as a dedicated protein design model but also as a versatile tool for a wide array of protein engineering applications.

**Fig. 3:**
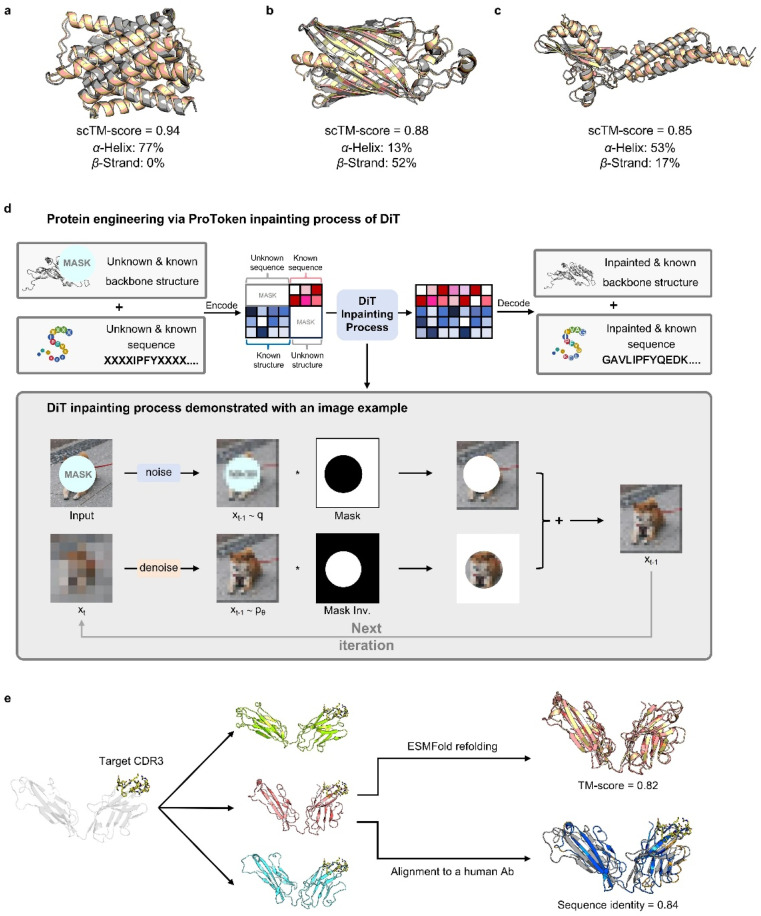
Showcasing protein sequence and structure engineering through PT-DiT. (a–c) Co-generation of protein sequences and structures for different protein folds: the α-helix-dominated structure (a), β-strand-dominated structure (b), and complex folding structure (c). (d) The inpainting workflow of PT-DiT, where a partial sequence or structure is inputted, encoded into latent space, inpainted through the DiT inpainting process, and then decoded into a full sequence and structure. (e) Structures of CDR3 grafting results, including ground truth (gray), generated structures (green, salmon, and cyan), and structural alignments to the ESMFold-refolded structure (wheat) and the ground truth structure (gray).

To illustrate PT-DiT's co-generation capabilities, we showcase three representative *de novo* generation cases featuring distinct geometries: helix bundles (Fig. 3a), β-barrels (Fig. 3b), and other complex folds (Fig. 3c). The TM-scores comparing the ESMFold-predicted structures (derived from the generated sequences) to the generated structures (scTM-scores in Fig. 3a–c) are 0.94, 0.88, and 0.85, respectively. These examples highlight PT-DiT's ability to concurrently design protein sequences and structures with diverse geometries, while preserving self-consistency between each generated sequence–structure pair.

Similar to training-free image editing, PT-DiT can perform “inpainting” on masked ProTokens or sequence embeddings by leveraging the unmasked regions (Fig. 3d). One notable application is contextual backbone design. As a proof of concept, we employed PT-DiT for CDR (complementary determining region) grafting (Fig. 3e). Given a specified CDR3 sequence and structure, PT-DiT simultaneously generates both antibody sequences and their corresponding structures. Validation with ESMFold reveals one generated sequence–structure pair that achieves a scTM-score of 0.82 and a sequence identity of 0.84 compared to a known human-derived antibody, while preserving the CDR3 conformation in the newly generated protein. We also showcase several examples of ligand-binding pocket scaffolding (Fig. S3†). These proof-of-concept results indicate that PT-DiT can design functional proteins that retain and integrate crucial functional motifs, underscoring its potential in functional protein engineering.

### Structure interpolation captures intermediate metastable states in protein dynamics

By pretraining PT-DiT, we learned a compact latent space for both ProToken and sequence embeddings, facilitating protein sequence and structure manipulation in a differentiable domain (Fig. S4†). Consequently, PT-DiT is able to interpolate between two metastable conformations of the same protein sequence, revealing cryptic intermediate states that are otherwise difficult to elucidate *via* standard protein dynamics. Given that PT-DiT is pretrained on a large set of foldable protein sequences and structures (see Methods) and learns a latent space assumed to follow a Gaussian distribution with a convex probabilistic density function, latent vectors interpolated between two metastable structures are expected to represent intermediate states with high probability and low free energy.^43^

**Fig. 4:**
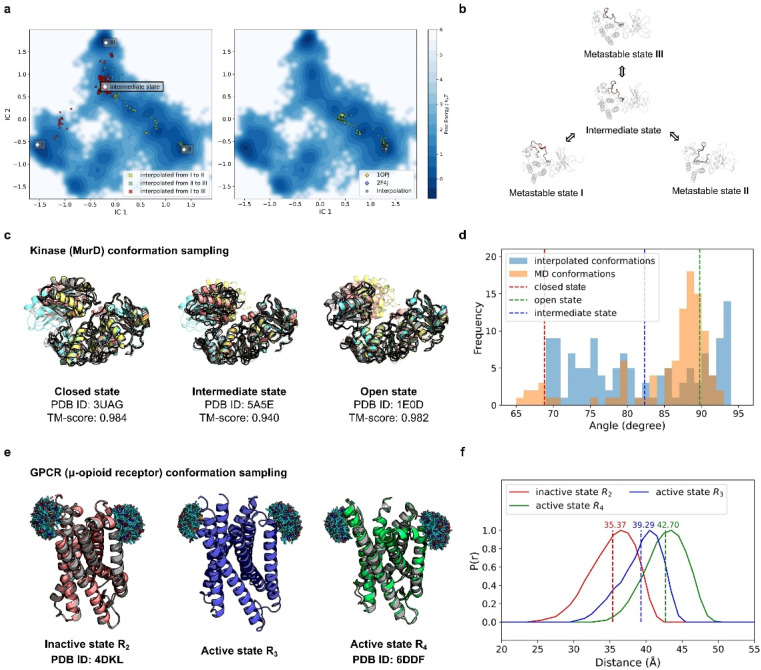
PT-DiT performance in metastable state sampling compared with MD simulations and experiments. (a) Free energy surface in TICA coordinates (IC1 and IC2) derived from MD simulations of Abl binding to imatinib. Conformational cluster centers (I, II, and III) are extracted from the trajectories, with scatter points showing the TICA projections of interpolated states: from I to II (yellow), II to III (green), and I to III (red) on the left. On the right, scatter points (green) represent the TICA projections of interpolated states between two experimentally resolved Abl structures (PDB IDs 1OPJ and 2F4J). (b) Protein structure illustration of metastable states during Abl binding to imatinib, sampled by PT-DiT, with the active loop region highlighted in salmon. (c) Closed, intermediate, and open states of MurD. Structures sampled by PT-DiT (colored yellow, salmon, and cyan for closed, intermediate, and open states, respectively) are superimposed with their corresponding crystal structures (gray). (d) Distribution of the collective variable defining the “hinge” motion between MurD domains, as observed in MD trajectories (orange) and interpolated structures (blue). The dashed red, green, and blue lines correspond to the experimentally resolved structures of the closed, intermediate, and open states, respectively. (e) Sampled structures of the inactive state R2 (salmon), and the active states R3 (blue) and R4 (green) of μOR. PT-DiT-generated R2 and R4 structures are superimposed with their corresponding crystal structures (gray). Ensembles of the attached fluorescent molecules (HO-1427) on residues R173 and R182 are displayed. (f) Predicted distribution of distances between fluorescent molecules in R2 (red), R3 (blue), and R4 (green), with average distances shown as dashed lines.

We benchmarked our interpolated conformations against a long-duration molecular dynamics (MD) simulation of Abelson tyrosine kinase (Abl) binding to the cancer drug imatinib,^44^ in which imatinib binding triggers a switch in the activation loop (A-loop). We analyzed the 10 ms MD trajectory using Time-lagged Independent Component Analysis (TICA),^45,46^ reducing the protein's conformational landscape to a two-dimensional space (Fig. 4a). Clustering these trajectories revealed three metastable states: I, II, and III (Fig. 4b).

Interpolation between these states was performed using PT-DiT, and the resulting intermediate structures were projected onto the TICA subspace (Fig. 4a, left). Between the three cluster centers, we identified an intermediate state with a low free energy region as seen from its location on the free energy surface (Fig. 4a and b). We also interpolated between the open (PDB ID 1OPJ) and closed (PDB ID 2F4J) X-ray crystal structures of Abl^44^ (Fig. 4a, right). The pseudo-trajectory in latent space traverses low free energy regions along the principal components defined by TICA, suggesting that the intermediate states sampled by PT-DiT are physically plausible.

Beyond aligning with the MD simulation trajectory, PT-DiT is designed to capture intermediate states that can be experimentally observed. We illustrate this by examining the transition of UDP-*N*-acetylmuramoyl-l-alanine:d-glutamate ligase (MurD) from its open conformation (PDB ID 1E0D)^47^ to its closed conformation (PDB ID 3UAG).^48^ The pseudo-trajectory generated by PT-DiT reveals domain 3 rotating around its hinge with domain 2 (Fig. 4c and S5†). Notably, one intermediate structure closely resembles the semi-open conformation identified by X-ray crystallography (TM-score = 0.94, PDB ID 5 A5E),^49^ proposed as a stable intermediate state during MurD's ligand-driven conformational change.

To further check the validity of the interpolated MurD conformations we introduce an angular collective variable, *θ* (details in Fig. S5†), based on previous MD studies of MurD's conformational dynamics.^50^ The distribution of *θ* values for the interpolated structures encompasses most of the range observed in both MD simulations and crystal structures (Fig. 4d). These findings indicate that PT-DiT can reliably generate experimentally validated intermediate conformations, thus offering a rapid alternative for sampling intermediate states of interest for experimental studies.

To further explore PT-DiT's capacity to elucidate hidden states in protein conformational transitions, we examined the μ-opioid receptor (μOR) system. Previous studies indicate that G-proteins bind to an open pocket formed by the outward movement of transmembrane helix 6 (TM6).^51^ Double electron–electron resonance (DEER) experiments classify the structural ensemble into four populations (R1–R4), distinguished by differing distances between TM6 and TM4. Both active and inactive states of μOR (PDB IDs 6DDF and 4DKL, respectively)^52,53^ exhibit an approximately 10 Å shift between TM6 and TM4. While R2 and R4 have been validated by X-ray crystallography (Fig. 4e, left and right), R3 remains unresolved. To address this question, we interpolated structures between R2 and R4 using PT-DiT, producing an intermediate conformation, R3′, whose TM4–TM6 distance closely matches the experimentally inferred value for R3 (Fig. 4f, ESI†). R3′ thus serves as a structural model for the unknown active state R3, providing a foundation for further biological analysis and design.

### Sequence interpolation discovers evolutionary and novel sequences between structural homologs

Sequence interpolation between structural homologs can also be performed in PT-DiT's latent space, analogous to structure interpolation. The trajectory between two sequences that stabilize a given structure can reveal novel variants capable of stabilizing the same fold.

**Fig. 5:**
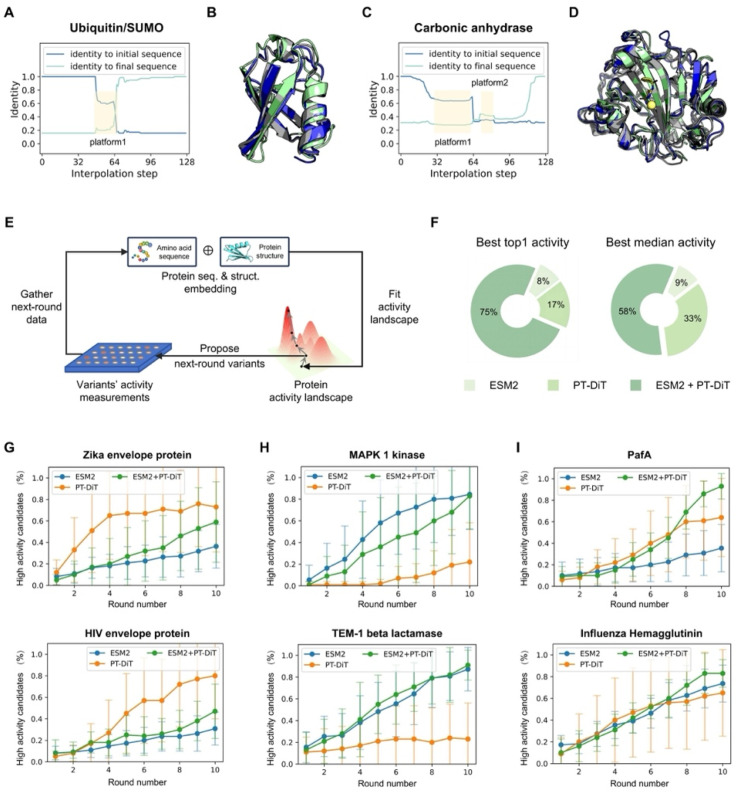
PT-DiT performance in evolution-like protein discovery and directed evolution. (A) Sequence identity of interpolated proteins compared to ubiquitin (PDB ID 1D3Z) and SUMO (PDB ID 1U4A). The sequence from platform1 shows a 94% identity with NEDD8 (PDB ID 3DBH). (B) Structural alignment of the rediscovered ubiquitin-like sequence from platform1 (gray) with structures of 1D3Z (cyan) and 1U4A (green). (C) Sequence identity of interpolated proteins with respect to two carbonic anhydrases (PDB IDs 3DCW and 3JXG) from different species, highlighting platform1 and platform2. (D) Structural alignment of platform1 and platform2 (gray) with structures of 3DCW (cyan) and 3JXG (green). The sequence of platform1 shows 96% identity with another carbonic anhydrase (PDB ID 1AZM), while platform2 represents a *de novo* sequence modeled on the carbonic anhydrase backbone. (E) Overview of directed evolution with PT-DiT based on an active learning algorithm. (F) Proportion of PT-DiT, ESM2, and their combination achieving the highest top activity and highest median activity in the proposed sequences, with each method using 10 rounds of active learning and 10 variants per round. (G–I) High-activity candidate percentages across 10 rounds of simulated directed evolution for different DMS datasets. Panels (G), (H–I) show the results for PT-DiT, ESM2, and the combination of PT-DiT and ESM2, respectively.

Small ubiquitin-like modifier (SUMO) proteins, which covalently bind to target proteins to modulate their functions, share structural similarities with ubiquitins but exhibit distinct functional outcomes. Despite having nearly identical backbone structures, human ubiquitin (PDB ID 1D3Z)^54^ and human SUMO-3 C47S (PDB ID 1U4A)^55^ share only 16% sequence identity (12 out of 76 residues), classifying them as remote homologs. To explore other potential remote homologs that preserve this common backbone, we performed interpolation in PT-DiT's latent space between 1D3Z and 1U4A. Throughout the interpolation pathway, the backbone structure remained largely unchanged while the sequence content evolved. Notably, sequence identity varied in a non-linear manner, forming a “platform” along the interpolation trajectory (Fig. 5A). One intermediate sequence from this platform (Fig. 5B, gray) has only 61% and 20% sequence identity to 1D3Z and 1U4A, respectively, but exhibits a 96% sequence identity to human NEDD8 (PDB ID 3DBH)^56^—another ubiquitin-like protein that shares the same structural scaffold. These findings underscore PT-DiT's ability to encode the full range of sequence variation that supports a common backbone structure.

We next investigated whether this interpolation strategy could produce *de novo* sequences. Carbonic anhydrases (EC 4.2.1.1), which catalyze the reversible conversion of carbon dioxide and water into carbonic acid, play a vital role in regulating pH and the acid–base balance in biological systems. Specifically, we performed interpolation between a human carbonic anhydrase (PDB ID 3DCW)^57^ and a homolog from *Mus musculus* (PDB ID 3JXG),^58^ which share 31% sequence identity. The resulting trajectory showed uneven changes, forming two “stable sequences” (Fig. 5C). The first stable sequence aligns with human carbonic anhydrase I (PDB ID 1J9W)^59^ at 93% sequence identity. Intriguingly, the second sequence has no close natural homologs in the UniRef100 database^60^ (maximum sequence identity 64%). Nonetheless, both ESMFold and AlphaFold3 confidently fold this sequence into the same backbone as 3DCW and 3JXG (Fig. S6†), demonstrating its ability to stabilize the carbonic anhydrase scaffold. These findings show that PT-DiT's unified sequence–structure representation not only reveals potential remote homologs but also generates novel sequences, presenting a fresh paradigm for protein design.

### Directed evolution in compact protein sequence–structure space

Controlled evolution has proven valuable for protein functional engineering,^61,62^ but exploring the vast mutation space of protein sequences can be both time-consuming and costly. As demonstrated above, the latent space of PT-DiT offers a compact and informative representation of both protein structures and sequences, potentially reducing the space to search in and facilitating controlled evolution for function enhancement.

Here, we use 12 Deep Mutational Scanning (DMS) datasets demonstrated in EVOLVEpro^33^ to simulate directed evolution, focusing on how different protein representations influence evolutionary outcomes. Unlike protein language models (PLMs) that rely solely on sequence- or MSA-based representations, we benchmark PT-DiT embeddings—which learn from both the structure and sequence independently—against the ESM2-15B baseline within EVOLVEpro. This integrated approach is expected to incorporate more structural information into the latent vector space. We also concatenate PT-DiT embeddings with ESM2-15B embeddings to form unified embeddings and explore whether these different embeddings complement or conflict with each other.

The results indicate that PT-DiT embeddings perform comparably to ESM2-15B embeddings across all 12 DMS datasets (Fig. 5F–I and S7A–F†). For top-1 activity predictions, the unified embeddings achieve 75% of the best outcomes, while PT-DiT and ESM2-15B respectively yield 17% and 8%. When evaluating median activity, unified embeddings achieve 58%, PT-DiT 33%, and ESM2-15B 9%, respectively. Notably, PT-DiT excels in systems involving signal transduction (Fig. S7E†), viral replication (Fig. 5G), and enzymatic activities (Fig. 5I), where incorporating structural information is thought to be crucial for capturing the functional impact of mutations.

Although PT-DiT may underperform ESM2-15B in certain high-activity prediction scenarios, combining embeddings from both models does not compromise overall accuracy (Fig. 5H). In contrast, this combined strategy improves the proposal rate for high-activity variants (Fig. 5I), enhancing both median and top activities among the predicted variants. Given that ESM2-15B embeddings in EVOLVEpro are 5120-dimensional whereas PT-DiT embeddings are only 40-dimensional, these findings underscore the compactness and efficacy of PT-DiT embeddings, suggesting the latter to be both more tractable and better suited for downstream biophysical applications.

## Conclusions

By compressing 3D structures of proteins into amino-acid-like sequences, ProTokens have demonstrated their ability to bridge the gap between sequence and structure modalities in protein engineering (Fig. S8†). As “machine-learned” amino acids, ProTokens represent an expanded vocabulary of natural amino acids, striking a better balance between informativeness and compactness. ProTokens are nearly as compact as amino acids; a vocabulary of a mere 512 terms is sufficient to encode and refold almost all existing protein folds, including novel and rare folds typically identified as “dark” clusters^63^ (details in ESI, Fig. S9†). On the other hand, ProTokens are more informative than amino acids. They can not only be easily “folded” (decoded) back into original structures with a lightweight module, without the need for sequence alignment and structural templates (whereas folding with amino acids requires cumbersome sequence processing and folding algorithms), but can also distinguish between different conformational states of a protein. These states are often degenerate in the representation of amino acid sequences, making ProTokens a finer descriptor of structures and thus more closely related to the functions of proteins.

From the perspective of the pretraining model of proteins, sequences and structures are interconnected in two fundamental ways: structures are linearized into sequences of neural amino acids, while from another viewpoint, amino acids represent nature's own strategy for compressing and storing protein structures. Consequently, leveraging ProTokens enables the convenient training of this foundational model, PT-DiT, to capture the joint probability of sequences and structures. Through generative pre-training, PT-DiT not only facilitates the co-design of matching sequences and structures but also supports the tailored design of proteins based on specific contexts, enhancing its versatility as a tool for protein engineering.

Furthermore, PT-DiT generates latent representations of proteins (both sequences and structures) that form a more structured space, facilitating higher-level abstractions of the protein universe. We discovered that perturbing, interpolating, and evolving proteins in this latent space give rise to numerous applications. For example, interpolating between two conformations of the same protein helps identify potential intermediate states of protein dynamics. These encrypted conformational states may offer new insights into the possible mechanisms by which proteins perform their functions. Simultaneously, we observed that interpolating between two sequences sharing a common backbone structure aids in identifying other sequences that can also stabilize it. Rediscovering naturally occurring sequences may reveal evolutionary pathways, while the discovery of *de novo* sequences offers a new methodology for protein design. Furthermore, the compact and organized latent space proves suitable for evolving proteins to enhance their activities toward specific objectives. Through adaptively learning activity profiles of mutations across multiple rounds of experimental feedback, PT-DiT yields high-activity candidates among both selected and proposed variants (more details in ESI, Fig. S10 and S11†).

The experiments performed in this study showed that unifying sequence and structure modalities of proteins using ProTokens is highly beneficial. From a physical standpoint, we verify the hypothesis that while the spaces of protein structures are inherently large, the metastable states can be considered countable and discretizable. We further developed efficient algorithms to discretize and represent these metastable states as machine-learned neural amino acids. Technically, compared to redundant and complex structural representations such as atomic coordinates or surface meshes, ProTokens provide a more compact and regular format for protein structures, making them more machine-friendly and suitable for computational models in structural biology. Furthermore, this amino-acid-like representation naturally integrates with protein sequences, enabling joint modeling of both the sequence and structure.

By mapping the structure to ProTokens, we have demonstrated that structure comparison and protein design benefit significantly from the compactness and convenience ProTokens provide. Moreover, by utilizing ProTokens in a latent space that organizes proteins to accommodate a generative objective, applications such as directed interpolation and evolution naturally emerge, significantly facilitating protein engineering. However, there are certain limitations to our approach. First, ProTokens, while effective, are far from an optimal code for proteins. In practice, we have observed a “degeneracy” in which highly similar structures may be encoded into different ProToken sequences. This degeneracy can hinder several downstream applications by increasing model confusion and suggests the potential for a more compact representation and further compression. Second, the exploration of PT-DiT's possible applications is still in its early stages. Many promising avenues remain to be explored, such as large-scale structural sampling, evolutionary analysis, functional annotation, and performing Bayesian optimization of protein physicochemical properties based on latent space representations. Future efforts will focus on improving compression rates and expanding the scope of applications for PT-DiT.

## Methods

### Datasets

In this study, we curated multiple datasets for a variety of tasks, enabling us to benchmark both ProTokens' protein reconstruction capabilities and PT-DiT's versatile downstream applications. Here, we describe the reconstruction validation datasets, molecular dynamics (MD) trajectory datasets, and experimental datasets in detail. Additional information is provided in the ESI.†

#### The reconstruction validation datasets

To prevent validation data leakage from the training set, we curated four single-domain validation datasets. The first comprises structures from the RCSB database released between October 13, 2021 and March 15, 2022, as well as CAMEO targets released between October 16, 2021 and February 12, 2022. These proteins were filtered at 40% sequence identity and restricted to sequences with fewer than 1536 residues, yielding 513 ground truth structures. The second and third datasets come from CASP14 and CASP15, respectively, encompassing all single-domain proteins with experimentally determined structures—87 proteins for CASP14 and 45 for CASP15. The fourth dataset is derived from the AFDB database, which contains 711 705 “dark clusters” identified by Foldseek, potentially enriched with novel protein folds. Following Foldseek's data processing pipeline, we selected 33 842 clusters with average AlphaFold2 prediction confidence scores (pLDDT) greater than 90, retaining only the highest-confidence member from each cluster. These dark cluster structures can be accessed at the AlphaFold DB website (https://alphafold.ebi.ac.uk/), and a complete list of names is available at https://afdb-cluster.steineggerlab.workers.dev/.

The multi-domain reconstruction task is evaluated using the CASP14 and CASP15 multi-domain datasets, which include domain annotations published on the CASP website. This process yields 17 multi-domain samples for CASP14 and 13 for CASP15. The multimer reconstruction task is assessed using the benchmark dataset of AF2Complex. More details of multi-domain and multimer datasets can be found in the ESI.†

#### The MD trajectory dataset of Abl and MurD

We used published results from unguided molecular dynamics (MD) simulations on Anton^64^ to generate the trajectory of Abl tyrosine kinase binding to imatinib. These simulations revealed an unexpected local instability in the C-terminal lobe of Abl during drug binding, making the system well-suited for sampling metastable conformations with PT-DiT. Conformations extracted from trajectories of 100 μs, 100 μs, 3 μs, 2.6 μs, 1.7 μs, and 1.7 μs were employed for time-lagged independent component analysis (TICA) and structure clustering, saving frames at 1000 ps intervals with a simulation timestep of 2.5 fs.

For MurD, we used MD trajectories from a previous study in which simulations were performed with Amber ff14SB under NAMD, using a 2 fs timestep for a total of 200 ns. All conformations from this trajectory were included in the variable analysis; during the course of the simulation, MurD transitioned from its closed to open state. The collective variable for MurD was defined based on the centers of mass of three residue selections: residues 120–230, 230–299, and 299–437.

#### Experimental dataset

The experimental data used in this study comprise several experimentally resolved protein structures and 12 Deep Mutational Scanning (DMS) datasets. The experimentally determined structures include (1) the active and inactive conformations of Abl, (2) open, closed, and intermediate conformations of MurD, (3) the active and inactive states of the μ-opioid receptor, (4) remote homolog structures of ubiquitin and SUMO, and (5) remote homolog structures of carbonic anhydrases. All structures can be accessed in the RCSB Protein Data Bank (RCSB PDB) under their respective PDB IDs. The DMS datasets encompass 12 experimental investigations targeting DNA-binding proteins, RNA-binding proteins, viral spike proteins, RNA-guided nucleases, and kinases. These same datasets have also been employed by EVOLVEpro to benchmark downstream biological tasks. More details can be found in Table S1.†

### ProTokens

#### Overview

ProTokens are residue-wise discrete tokens for encoding of proteins' metastable states. In the landscape theory,^65^ a metastable state is a basin on the free-energy surface that remains stable over an observation time (*τ*_obs_) longer than the local relaxation time (*τ*_relax_) but shorter than the state's overall lifetime (*τ*_life_). Under these conditions, the protein primarily explores local fluctuations within that basin rather than transitioning to others. By analogy, amino acids can be considered as a highly compressed set of tokens that define the folded state at *τ*_obs_ ≈ *τ*_fold_, where *τ*_fold_ is the folding/unfolding timescale of a protein (Fig. S12†). Our ProTokens extend this concept to finer timescales, producing a rich yet still discrete representation that can capture functional conformational variations and be reliably mapped back to 3D coordinates. Building on ProTokens, a unified perspective is achieved for understanding the sequence and structure modalities in protein science, which are traditionally viewed as separate domains. Detailed discussion of probabilistic tokenization theory can be found in the ESI.†

#### Model architecture

A VQ-VAE based network named ProToken Distiller is designed to tokenize the metastable structure **x** of proteins. The ProToken Distiller includes three main components: the encoder *f*_*θ*_, the tokenizer *h*_*θ*_, and the decoder *g*_*ϕ*_ (details in ESI, Fig. S13†).

The encoder *f*_*θ*_ is a parameterized SE(3)-invariant mapping that transforms a protein structure **x** with *N*_res_ residues into a *d*_s_ dimensional single representation 
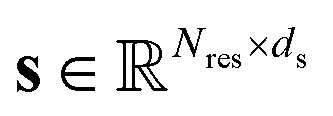
 and a *d*_p_ dimensional pair representation 
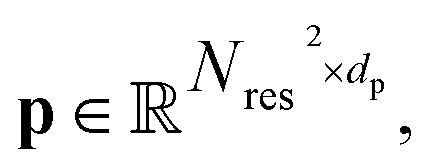
 derived from its distance matrix and backbone dihedrals. Inspired by the EvoFormer and structure module in AlphaFold2, we form a scalable “sandwich-like” transformer module comprising 2, 4, and 2 layers of EvoFormer, ResiDual transformer, and EvoFormer, respectively. These layers are designed to update single representations and pair representations, and co-update single and pair representations sequentially. Subsequently, a structure module is utilized to enhance the encoder's structure-awareness by aggregating information from the processed **s**, **p** and the raw structure **x**, and finally output a *d* dimensional representation 
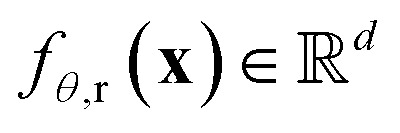
 for each residue *r* (1 ≤ *r* ≤ *N*_res_).

For the tokenizer *h*_*θ*_, we employ vector quantization (VQ)^23^ techniques commonly used in image tokenization. The VQ module dynamically maintains and updates a “codebook” {**c**_*i*_}, 
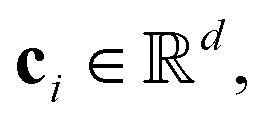
 which serves as cluster centers in the vector space. Each input vector *f*_*θ*,r_(**x**) is assigned to the nearest code **c**_*i*_ in the codebook *via* a nearest neighbor search:1
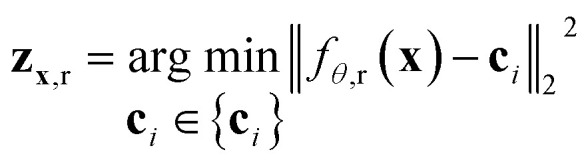
Here, the ProToken for residue *r* is defined as the code **z**_**x**,r_ = **c**_*i*_ and the corresponding “token index” *i*. The decoder *g*_*ϕ*_(**z**) maps the ProToken sequence **z**_**x**_ = (**z**_**x**,1_, **z**_**x**,2_, …, **z**_**x**,*N*_res__) back to the input structure, such that *g*_*ϕ*_(**z**_**x**_) ≈ **x**. Similarly, the decoder consists of a “sandwich-like” transformer of identical size to the encoder, followed by a structure module that “folds” the structure from the single and pair representations.

#### Training of ProTokens

The training objective is simply to reconstruct the structure from the encoded ProTokens, *i.e.*, *g*_*ϕ*_(*h*_*θ*_(*f*_*θ*_(**x**))) ≈ **x**. Since reconstructing the structure from ProTokens closely resembles protein folding, we adopted the Frame Aligned Point Error (FAPE)^18^ and the structure violation loss used in AlphaFold2 as the reconstruction loss. Additionally, as in standard VQ models, a commitment loss is implemented to regularize the codebook and embedding vectors.

In most protein engineering scenarios, it is essential to represent protein metastable states defined on a functional timescale, where *τ*_obs_ = *τ*_func_ < *τ*_fold_. To this end, we use structures from the RCSB database as the training set, as they are often regarded as functionally relevant conformations of proteins. Metastability implies that the structural ensemble {**x**}_*τ*≪*τ*_obs__ should be tokenized into the same set of ProTokens, with irrelevant fluctuations at *τ* ≪ *τ*_obs_ treated as noise. To mimic the distribution of structural fluctuations, we augment the structural data by applying perturbations (details in the ESI†). Additionally, we apply an alignment loss^27^ to constrain the embedding vectors of two structures **x** and **x**′ belonging to the same metastable state:2
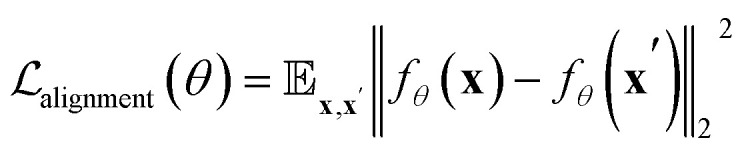


To further optimize the representation, a uniformity loss is introduced to encourage the code distribution to be as uniform as possible, ensuring efficient use of the latent space,3
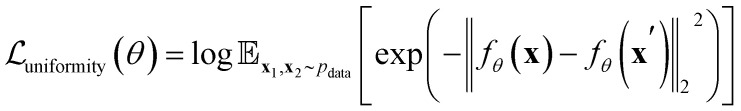


Details on the design rationale of the models and training algorithms are provided in the ESI.†

#### PT-score algorithm

Similar to Foldseek,^26^ to obtain the substitution matrix for homologous structural domains, we performed extensive data augmentation on the training set. Specifically, we utilized AlphaFold2, using the ground truth structure of the protein as a template, without adding multiple sequence alignments (MSAs) and enabling dropout during structure prediction. We then filtered out the high quality perturbation samples where the TM-score of the perturbed structure and true structure is above 0.8. The Local Distance Difference Test (LDDT) between the predicted and the true structures was conducted and residues with LDDT scores greater than 80 were selected. The corresponding structural code pairs were counted to compute the BLOSUM matrix. In parallel, we used the Needleman–Wunsch algorithm to calculate the similarity and alignment of two ProToken representations, yielding a PT-score. The Needleman–Wunsch algorithm was implemented using a custom Python package.

### PT-DiT

#### Training of PT-DiT

Based on ProTokens, we represent proteins with vectors **z** = (**z**_**x**_, **z**_**s**_), where **z**_**x**_ denotes residue-wise ProToken embeddings (dim = 32) distilled from backbone structures, as defined in eqn (1), and **z**_**s**_ represents residue-wise amino acid embeddings (dim = 8), reduced *via* principal component analysis (PCA) from ESM2 and AlphaFold2 embeddings. This embedding strategy can be formulated as the Cartesian product of ProTokens (tokenized backbone structures) and sequences, forming a homogeneous and interactable space for both sequence and structure modalities. Thus, the probabilistic model *p*(**z**_**x**_, **z**_**s**_) captures the joint likelihood of sequences and structures. After **z**_**x**_, **z**_**s**_ are generated according to *p*(**z**_**x**_, **z**_**s**_), the backbone coordinates of the protein are decoded from **z**_**x**_ using the ProToken decoder, while the protein sequence is decoded from **z**_**s**_*via* nearest-neighbor search.

By virtue of the regularity of the compact vector space *p*(**z**_**x**_, **z**_**s**_), adapting diffusion models to generate proteins is as straightforward as generating images or videos. We employ standard denoising diffusion probabilistic models (DDPMs),^66^ where the noise prediction model *ε*^θ^(**z**_*t*_, *t*) is trained to reverse a Markov diffusion process 
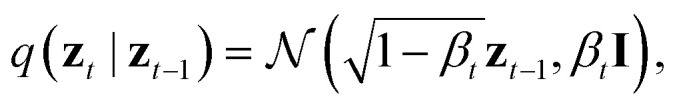
 which is equivalently defined by the following transition function of probability,
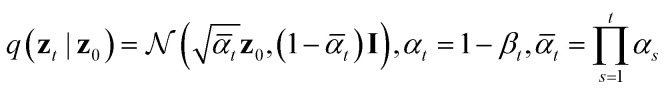


The architecture of *ε*_*θ*_(**z**_*t*_, *t*) follows that of diffusion transformers, utilizing 24 transformer layers with a hidden size of 512 to predict noise from the perturbed protein embedding **z**_*t*_. The following “noise-matching” loss is defined for training,
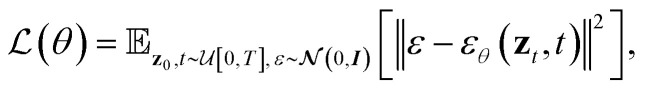

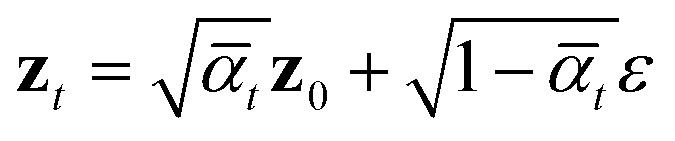


After training, the (**x**, **s**) pair can be generated *via* the ancestral sampling scheme of DDPM, which samples the backward diffusion process, defined as:
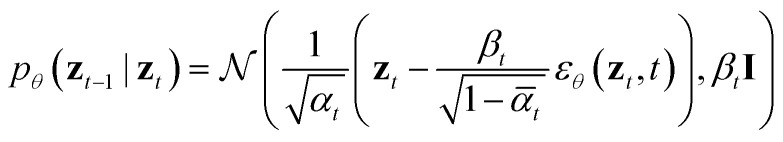


Alternatively, other samplers based on stochastic differential equations or ordinary differential equations can also be applied.^25^ The model architecture closely follows that of diffusion transformers, and state-of-the-art image diffusion models proven to scale effectively in image generation. Moreover, PT-DiT is highly adaptable to the latest advancements in both training and sampling techniques developed for image diffusion models, as we made minimal modifications to the model architecture and training algorithms.

#### Algorithms for protein engineering

By unifying the perspectives of sequences and structures and projecting these two modalities into a joint embedding space, various tasks in protein engineering can be interpreted as an “inpainting” problem.^24^ Inverse folding involves filling in sequence embeddings with designated structure embeddings, while structure prediction or sampling corresponds to generating structure embeddings using sequence embeddings as context. Furthermore, contextual design can be achieved by inpainting the remaining sequences and structures, given that the sequences and/or structures of part of the residues (often functional regions) are provided. Many methodologies have been developed to leverage pre-trained image diffusion models for image inpainting, most of which can be directly adapted to PT-DiT. In our experiments, we applied the RePaint algorithm,^24^ where each step involves harmonizing the context and non-context regions under specific noise levels.

By leveraging the RePaint strategy, although PT-DiT is trained to model the joint probability distribution of entire protein sequences and structures, it can be directly applied to many conditional generation tasks in protein engineering without the need for additional training or fine-tuning. Mathematically, PT-DiT combined with RePaint algorithms solves the conditional generation problem *p*(**z**_**x**_, **z**_**s**_|**z**^c^_**x**_, **z**^c^_**s**_), where **z**^c^_**x**_ and **z**^c^_**s**_ represent context ProTokens and amino acid embeddings, respectively. For example, in the inverse folding task, **z**^c^_**x**_ = **z**_**x**_ corresponds to the full ProToken sequence derived from input backbone structures, while **z**^c^_**s**_ = *∅*. For scaffolding, **z**^c^_**x**_, **z**^c^_**s**_ encode the structure and sequence information of the functional site that is preserved during generation.

To demonstrate how PT-DiT with the RePaint algorithm can graft specific functional motifs onto a different scaffold, we use the CDR3 of antibodies as an example. The procedure proceeds as follows: as shown in Fig. 3e, we first crop the CDR3 from an antibody heavy chain with a known structure (PDB ID 5JXE).^67^ Since loop-like conformations are ubiquitous in protein structures, merely specifying a CDR3 loop as context cannot ensure the generated structures belonging to the family of antibodies which exhibit specific structural constraints. Therefore, we need to precondition the contextual ProTokens towards antibody-like structures. Specifically, we first select a human germline structure as the template, and then replace its CDR3 region with the to-be-grafted loop by superimposition. The backbone of this artificial grafted structure is encoded into backbone tokens, among which the CDR3 loop is cropped and set as context along with its sequence. The lengths of the flanking FWRs and CDRs can be sampled according to the distribution of the human germlines, while we set them the same as in 5JXE for simplicity. Through inpainting sampling, we can obtain ProTokens that can be decoded to all-atom structures containing the target CDR3 loop as well as the amino acid sequence for the entire chain.

#### Definition of latent representations

Mathematically, the forward and backward diffusion processes of DDPMs can be continuously formulated as the following forward and backward stochastic differential equations (SDEs):^25^
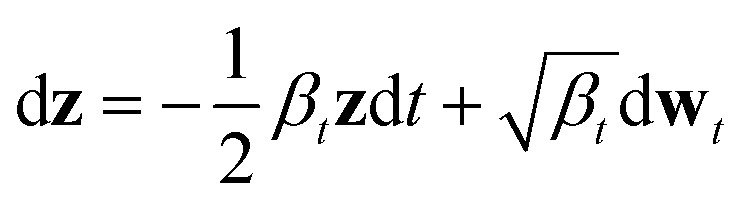

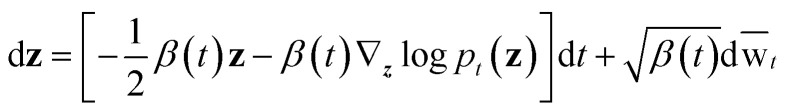
where *β*_*t*_ defines the forward Markov chain of DDPM 
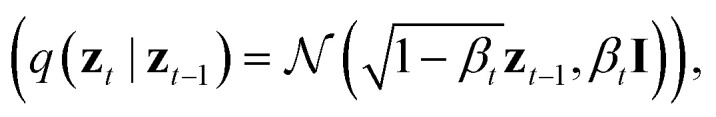
**w**_*t*_ denotes a standard Brownian motion, and ∇_**z**_ log *p*_*t*_(**z**) represents the score function of marginal distribution at *t*, which is approximated by the neural networks. Furthermore, these SDEs can be equivalently transformed into a probability flow ordinary differential equation (PF-ODE),^68^ which conserves the marginal distribution of **z**(*t*) at each time step *t* ∈ [0, 1],
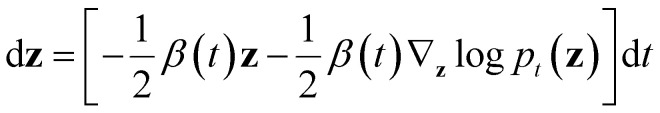


Therefore, a DDPM is also a latent generative model, where a deterministic one-to-one mapping between the input vectors **z**(*t* = 0) and the latent vectors **z**(*t* = 1) is constructed by solving the initial value problem (IVP) using any black-box ODE solvers, such as Euler and Runge–Kutta solvers.

The strategy used to interpolate between two proteins in latent space is straightforward. After mapping protein A and protein B (represented as **z**_A_(0) and **z**_B_(0)) into latent vectors (using the explicit 4^th^ order Runge–Kutta method), **z**_A_(1) and **z**_B_(1), we obtain their intermediate latent vectors *via* linear interpolation:**z**_*λ*_(1) = (1 − *λ*)**z**_A_(1) + *λ***z**_B_(1)

Subsequently, the intermediate proteins (**z**_*λ*_(0)) are obtained by solving the backward IVP.

### Statistical analysis

#### rTM-score and rLDDT

The TM-score is widely used to evaluate the fold similarity between two protein structures. Two protein folds with a TM-score above 0.5 are usually considered as the same fold. To evaluate the structure decoded by the ProTokens of the protein with its original ground truth structure, we use this score to show the structure difference and specially call it the rTM-score. The Local Distance Different Test (LDDT) is another method commonly used for evaluating the residue-wise structure difference of two proteins. The LDDT score between the structure decoded from the ProTokens and its original ground truth structure (rLDDT) is calculated to show explicit structure reconstruction performance of ProToken. The rTM-score is calculated using the official code (https://zhanggroup.org/TM-score/). rLDDT is calculated with custom python code.

#### scTM-score

As a co-generation method of the protein structure and sequence pair, we use the TM-score matrix between the ESMFold predicted structure of the generated sequence and the generated structure to evaluate the self-consistency of the structure and sequence pair and specifically call it the scTM-score for ease of recognition.

## Data availability

The training sets used in this study are publicly available at https://ftp.cbi.pku.edu.cn/psp/. All the information for experimental datasets is listed in Table S1.† All data used and showed in this study are available at the https://doi.org/10.17605/OSF.IO/E9T8W repository.^69^ The ProToken and PT-DiT code is available at https://github.com/issacAzazel/ProToken under Apache 2.0 license. A Colab notebook for ProToken encoding and decoding process is provided at https://colab.research.google.com/drive/15bBbfa7WigruoME089cSfE242K1MvRGz for ease of use. We also provide several notebooks for downstream tasks mentioned above like directed evolution (https://github.com/issacAzazel/ProToken/blob/main/example_scripts/latent_space.ipynb), *de novo* design (https://github.com/issacAzazel/ProToken/blob/main/example_scripts/de_novo_design.ipynb), CDR3 grafting (https://github.com/issacAzazel/ProToken/blob/main/example_scripts/repaint.ipynb), and conformation sampling for ease of use.

## Author contributions

Y. Gao, J. Zhang, and S. Feng developed the overall concepts in the paper and supervised the project. X. Lin, Z. Chen, and Y. Li developed and benchmarked the model and/or contributed to the code. Z. Chen, X. Lin, and Z. Ma performed data collection and analysis. X. Lin, Z. Chen, and Y. Li wrote the initial draft of the manuscript. All authors contributed ideas to the work and assisted in manuscript editing and revision.

## Conflicts of interest

The authors declare that they have no competing interests.

## Supplementary Material

SC-OLF-D5SC02055G-s001
