# Supplementary material for: Unifying sequence-structure coding for advanced protein engineering via a multimodal diffusion transformer
Source: Chem Sci. 2025 May 15;16(24):11087–102. doi: 10.1039/d5sc02055g (PMC12096517; doi:10.1039/d5sc02055g)
Supplement: SC-016-D5SC02055G-s001 [file SC-016-D5SC02055G-s001.pdf]

Supplementary Information (SI) for Chemical Science.
This journal is © The Royal Society of Chemistry 2025

Supplementary Materials for

Unifying Sequence-Structure Coding for Advanced Protein Engineering via a Multimodal Diffusion Transformer

Xiaohan Lin *et al.*

*Corresponding author. Email: fengsh@cpl.ac.cn; jzhang@cpl.ac.cn; gaoyq@pku.edu.cn

This PDF file includes:
Supplementary Text
Figs. S1 to S13
Tables S1
References (1 to 55)

Supplementary Text

Pitfalls behind Transform Representation of Protein 3D Structures

After transforming the spatial coordinates of metastable structures into SE(3)-invariant (continuous or discrete) representations like ProTokens, one may apply them for downstream tasks related to protein structures. However, we reveal that high risk exists if the transformed representations are not optimized or implemented with caveats, and summarize several potential pitfalls of learning and applying a transformed representation. Awareness of these issues are reflected in the specifically designed model components and training objectives which will be elaborated in the following sections.

A. The space of the transformed representation should be compact.

Taking ProTokens of a N_{res}-long protein as example, the real-valued token embeddings take the shape of (N_{res}, d). If $d \gg 3\bar{N}_{atom}$ (where $\bar{N}_{atom}$ stands for the average number of atoms per residue), diffusion in the transformed embedding space will be less efficient (both in terms of data and training) than spatial diffusion approaches like RFDiffusion and AlphaFold3(*1*, *2*), provided that the likelihood of the model decays exponentially with the dimensionality of the representation.

Besides, the number of all possible combinations of ProTokens grows combinatorially with respect to the vocabulary size K. Since the number of foldable protein structures (as well as functionally relevant metastable states) is unlikely to exceed $20^{N_{res}}$, the reasonable number of tokens used during training should lie in the range between tens to a few hundreds.

To extract compact ProTokens, we design a *Compressing* module in the *ProToken Distiller* to properly compress the ProToken embeddings lengthwise or depth-wise. Besides, we derive a variational information bottleneck (VIB)(*3*) to quantify the necessity of ProTokens , and the vocabulary size K is optimized with respect to the VIB loss by *variational clustering* during training (Eq. S4.6).

B. The transformed representation should be robust against intrinsic structure fluctuations.

In terms of protein physics, fluctuations are intrinsic to metastable protein structures, which may cause subtle structure changes but do not alter the function. One particular concern of a learned structural representation is that the *robustness* of the yielded embeddings and tokens against subtle structure perturbation may not be guaranteed (fig. S1), while the susceptibility to intrinsic fluctuations will be harmful to downstream tasks such as function predictions.

To alleviate this issue, we introduce adversarial examples by adding physics-informed fluctuations to any input structure, and adversarially train the model to behave robustly to the *adversarial attacks*. Besides, we also train a *Deduplicator* module to relax fluctuated structures within a

metastable ensemble towards a single stable representative structure, and collapse the embeddings of the fluctuated structures.

C. Existence vs. uniqueness: The degeneracy of transformed representation should be addressed.

Noteworthy, the *uniqueness* of a learned ProToken corresponding to a certain structure x cannot be guaranteed through data-driven training. *Duplicate* or degenerate ProTokens may exist that can be decoded to (almost) the same structure (fig. S1). Degeneracy is particularly poisonous when ProTokens are used for maximum likelihood estimation (MLE) of protein structures by generative models such as auto-regressive and diffusion models. To be more specific, after transforming the representation of protein structure x to ProToken z, which can be detokenized back via a decoder $x = g_\phi(z)$, the (log-)likelihood of the structure x should be computed in the following way as in latent generative models(*4*, *5*),

$$\log p(x) = \log \int p(z) p_\phi(x|z) dz = \log \sum_{z_i \in Z(x;\phi)} P(z_i) \#(S1.1)$$

where $p_\phi(x|z) = \delta(g_\phi(z), x)$ is defined as "*duplicate distribution*", which consists of all ProTokens $\{z\}$ that can be decoded back to x through the decoder. Since z is discrete, integration of $p_\phi(x|z)p(z)$ over z is equivalent to the summation of $P(z_i)$ over a finite and countable *duplicate set* $Z(x;\phi)$.

Since we barely have information about $Z(x;\phi)$ a *priori*, the exact likelihood in Eq. S1.1 is hard to compute in practice. To address this issue, we derive computationally efficient lower bounds (Eq. S3.7 & Eq. S3.10) for the likelihood and develop a *Duplicator* module to explore and expand the duplicate set to reduce the bias and variance for likelihood estimation.

Probabilistic Tokenization Theory of Protein 3D Structure

Although the structure of a protein can be continuously changed in the Cartesian coordinate space, the set of its metastable states are countable, hence, can be well-defined discretely according to the landscape theory(*6*, *7*). Specifically, the definition of metastable states depends on the observation timescale τ_{obs}: A metastable state can be only defined if $\tau_{rlx} < \tau_{obs} < \tau_{life}$. According to the landscape theory, the smaller τ_{obs} is, the larger amount of metastable states can be defined (fig. S12). In a limit case, when τ_{obs} is comparable to the (un-)folding timescale (τ_{fold}) of a protein, most proteins are known to exhibit a two-state kinetics between the folded and unfolded states, hence, the only folded metastable state can be well defined by the amino-acid sequence of the protein.

Thanks to the metastability, a continuous distribution of function-related protein structures can now be reasonably factored into discrete and continuous parts,

$$\int p(x) dx = \int p(x \mid z) p(z) dz dx = \int p(\epsilon \mid z(x)) p(z) dz d\epsilon \#(S2.1)$$

where the discrete part z stands for the metastable state, $p(z)$ is a discrete distribution specifying the number of metastable states which $x \sim p(x)$ consists of, and the continuous part $p(\epsilon | z(x))$ covers the intrinsic structure fluctuations within this metastable state. The first equation holds because z is a deterministic function of x.

Equation S2.1 is the foundation of our probabilistic tokenization theory for protein structures. It justifies that a discrete prior (denoting the metastable states) for continuous protein structures is reasonable. It also implies that, if the metastable states are few (given a large τ_{obs}), ϵ should account for large and complicated variations within each state. On the contrary, if the metastable states are defined at a small timescale, ϵ is only responsible to very subtle structural variations within each state, but the number of z could quickly explode. Put it in other words, there is a trade-off between the compactness of z (i.e., the number of defined metastable states) and the informativeness of z (i.e., the residual intra-state variation that has to be explained by ϵ).

Inspired by the lattice model for proteins(*8*), it is plausible that the number of metastable states grow with the length of proteins, so we can assign a finite number of discrete states to each residue (namely, tokens), and the combination of these residue-wise tokens, define the overall state of the protein. From this perspective, amino acids can be considered a type of token, as Anfinsen's dogma states that protein structures can fold from their corresponding sequences. Thus, we refer to amino acids as "Anfinsen's tokens". Anfinsen's tokens are probabilistic in nature because they do not correspond to a single snapshot of protein 3D structure, but to all the folded structures with $\tau_{obs} \approx \tau_{fold}$ according to the Anfinsen's hypothesis. However, Anfinsen's tokens are extremely compact (with a small vocabulary size of only 20), thus, leaving the intrastate variations large and hard to estimate. That explains why conformational prediction based on amino acids is a tremendously challenging task. Due to the absence of an effective detokenization algorithm (although significant advance has been made since AlphaFold) which can trustworthily map back amino acids to the folded conformations of the protein, they are often not regarded as a 3D representation for protein structures.

Compared to amino acids (Anfinsen's tokens), tokenizing metastable states at finer timescale $\tau_{obs} < \tau_{fold}$ has several compelling advantages: i) more detailed changes in structures corresponding to functional switch can be described including alternative conformations; ii) tokens can strike good balance between being compact and being informative; iii) a more efficient detokenizer can be obtained to map back to the tokens. Conceptually, given a protein 3D structure x, we tokenize the metastable structure ensemble $\{x\}_{\tau \ll \tau_{obs}}$ associated with x into probabilistic, amino acid-like tokens (ProTokens), which can be detokenized back to conformations from the corresponding metastable state.

Algorithm Derived from the Probabilistic Tokenization Theory

A. Model Overview

Tokenizing metastable states associated with a given protein structure can be cast as a *conditional generative learning problem* as in invertible coarse graining(*9*). We design a deep neural network system, *ProToken Distiller*, to achieve this goal (fig. S13a).

1. Probabilistic conditional decoder

Specifically, we aim at constructing a metastable conformational distribution $p_\phi(x)$ and sampling from it according to a structure x, which can be achieved through a probabilistic conditional *Decoder* g_ϕ using the reparameterization trick as in VAE(*10*) or GAN(*11*),

$$x \sim p_\phi(x); \quad p_\phi(x)dx = g_\phi(\epsilon, z(x))d\epsilon \#(S3.1)$$

where ϵ is random noise from a known prior like Gaussian distribution, and $z(x)$ is the embedding of a tokenized x provided as the conditional information to the *Decoder*. According to the probabilistic tokenization theory (Eq. S2.1), the ProToken z specifies the identity of the metastable state, whereas ϵ accounts for the conformational fluctuations within the state.

The Decoder is a composite function consisting of a *Token Duplicator* module and a *Detokenizer* module: The *Token Duplicator* is responsible to expand and sample from the duplicate set in Eq. S1.1, whereas the *Detokenizer* module is a SE(3)-equivariant generative model which samples protein structures from a metastable ensemble corresponding to a given ProToken string.

The conditional embedding $z(x) = h_\theta \circ f_\theta(x)$ is obtained via a composite of *Encoder* f_θ and *Tokenizer* h_θ, which transforms the all-atom protein 3D structure x into SE3-invariant embeddings of discrete tokens.

2. SE(3)-invariant encoder

The *Encoder* f_θ comprises an SE(3)-invariant *Structure Encoder* module, and a *Deduplicator* module (fig. S13c). As explained, the *Deduplicator* module is introduced to improve the *robustness* of the yielded embeddings against intrinsic structure fluctuations. Given the separation of timescales of backbone and sidechain motions, the sidechain and backbone structures are encoded separately,

$$f_\theta(x) = f_\theta(x_{BB}, x_{SC}) \approx f_{\theta_1}(x_{BB}) \otimes f_{\theta_2}(x_{SC}) \#(S3.2)$$

where x_{BB}, x_{SC} denote the backbone and sidechain structures, respectively; and $\otimes$ denotes Cartesian product (i.e., concatenation of tensors). The resulting $f_\theta(x)$ is a continuous SE(3)-invariant embedding for the protein structure.

Considering that metastable structure ensembles can be reasonably represented by discrete tokens, we prepend a Tokenizer h_θ to the Encoder in order to *variationally cluster* (or discretize) $f_\theta(x)$ into quantized ProTokens.

3. Variational tokenizer

The Tokenizer $h_\theta = r_\theta \circ s_\theta$ discretizes the structural embeddings $f_\theta(x)$ into ProTokens $z(x)$ (fig. S13d), consisting of a composition of a *Clustering* module s_θ and a *Compressing* module r_θ. The *Clustering* module aggregates the continuous embedding learned by the Encoder into K clusters (i.e., "codes" or "tokens"), and each cluster is assigned with a *d*-dimensional vector as the cluster center (or token embedding). Since the backbone and sidechain embeddings are obtained through two independent tracks, the Clustering module also operates separately for backbone and sidechain embeddings,

$$z_{BB} = s_{\theta_1}(v_{BB});\ z_{SC} = s_{\theta_2}(v_{SC}) \# (S3.3)$$

z_{BB},z_{SC} denote the backbone and sidechain tokens, respectively. The ProToken for all-atom structure is then assembled by Cartesian product $z = z_{BB} \otimes z_{SC}$. Each ProToken has *dual representations* which are mutually mappable: one corresponds to the discrete cluster index, the other is the embedding of the cluster center which lies in a continuous vector space.

To make the clustering procedure end-to-end differentiable, we approximate the gradient flow with straight-through estimators(*12*, *13*) for backbone tokenization.

Noteworthy, there is a fundamental difference between the Clustering module and common VQ models such as VQ-VAE(*14*) or VQ-GAN(*15*): The clustering is performed *variationally*, that is, the number of alive codes should be as small as possible in order to tighten the variational information bottleneck, which contrasts sharply to state-of-the-art VQ training where a high usage of codes is usually preferred(*16*, *17*).

B. Decoder

The Decoder is a composite function consisting of a *Token Duplicator* module and a *Detokenizer* module.

1. Detokenizer

The Detokenizer module is a SE(3)-equivariant generative model which samples protein structures from a metastable ensemble corresponding to a given ProToken string. More specifically, the all-atom structures are generated in a factorized way by a backbone detokenizer g_{ϕ_1} and a sidechain detokenizer g_{ϕ_2}, respectively,

$$x \sim p_\phi(x_{BB},x_{SC}) = p_{\phi_1}(x_{BB})p_{\phi_2}(x_{SC} \mid x_{BB}) \# (S3.4)$$

$$p_{\phi_1}(x_{BB})dx_{BB} = g_{\phi_1}(\epsilon;z_{BB}))d\epsilon \# (S3.5)$$

$$p_{\phi_2}(x_{SC}|x_{BB})dx_{SC} = g_{\phi_2}(\epsilon;z_{SC},x_{BB})d\epsilon \# (S3.6)$$

where the backbone structure is first generated according to the $p_\phi(x_{BB})$ conditioned on the backbone tokens z_{BB}, followed by the generation of sidechains from $p(x_{SC}|x_{BB})$ conditioned on the backbone structure x_{BB} as well as the sidechain tokens z_{SC}.

Noteworthy, any unconditional backbone generative model (such as RFDiffusion(*1*)) can be adopted as an initializer for the backbone detokenizer further optimized through conditional fine-tuning. Similarly, any generative sidechain packer can be directly plugged-in (or fine-tuned) as the sidechain detokenizer. We implemented DLPacker(*18*) in this work and did not fine-tune it.

2. Token Duplicator

The duplicate set defined in Eq. S1.1 depends both on the volume of the ProToken space and the over-capacity of Decoder. The duplicate set associated with a less compact ProToken space, or a less invertible Decoder, intuitively tends to be larger, which is harmful to protein structure generation tasks as will be shown later.

Although the exact likelihood (Eq. S1.1) is hard to compute in practice, fortunately, we can develop proper lower bounds that can be conveniently used for maximum likelihood estimation of x through the transformed representation z.

The first lower bound (L_1) can be derived via the truncation trick. Given a (truncated) subset $\tilde{Z}(x) \subseteq Z(x;\phi)$,

$$\log p(x) = \log \sum_{z \in Z(x;\phi)} p(z_i) \geq \log \sum_{z \in \tilde{Z}(x)} p(z_i) := L_1(\tilde{Z}(x)) \quad (S3.7)$$

L_1 exhibits an important property that,

$$If\ \tilde{Z}_2(x) \supset \tilde{Z}_1(x),\ then\ L_1(\tilde{Z}_2(x)) \geq L_1(\tilde{Z}_1(x)) \quad (S3.8)$$

The equality holds if elements in the difference set, $\tilde{Z}_2(x) - \tilde{Z}_1(x)$, have zero probability density in total. L_1 is still inconvenient to compute in practice because the summation of probability is prior to logrithm. Thus, we further develop another lower bound (L_2) based on L_1 according to Jensen's inequality,

$$L_1(\tilde{Z}(x)) = \log \frac{\sum_{z \in \tilde{Z}(x)} p(z_i)}{|\tilde{Z}(x)|} + \log |\tilde{Z}(x)| \geq \frac{\sum_{z \in \tilde{Z}(x)} \log p(z_i)}{|\tilde{Z}(x)|} + \log |\tilde{Z}(x)| \quad (S3.9)$$

$$\log p(x) \geq L_1(\tilde{Z}(x)) \geq E_{z \sim \tilde{p}(z|x,\phi)} \log p(z) + \log |\tilde{Z}(x)| := L_2(\tilde{Z}(x)) \quad (S3.10)$$

Different from L_1, Eq. S3.8 allows us to perform minibatch optimization by unbiasedly sampling from a $\tilde{Z}(x)$ during training. Similarly, it also follows straightforwardly from Eq. S3.8 that the larger $|\tilde{Z}(x)|$ is, the tighter the lower bound L_2 is, indicating the importance of expanding the subset $\tilde{Z}(x)$.

The remaining issue is how to construct and sample from the duplicate (sub-)set. A naïve approach is to approximate the duplicate set by a singleton set, i.e., $\tilde{Z}(x) = \{f_\theta(x)\}$, which leads to a suboptimal truncation for L_1. Or one can determistically set $z = f_\theta(x)$ and estimate the expectation term in L_2, which leads to a highly biased and high-variance one-sample Monte Carlo estimator.

To circumvent these drawbacks, a *Token Duplicator* module, $q_\phi(z|x,g_\phi)$, is prepended to the Detokenizer, which is responsible to expand and sample from the duplicate subset $\tilde{Z}(x)$. In general, any conditional generative model which yields diverse ProTokens which can be decoded back to x via the Decoder can be used as $q_\phi(z|x,g_\phi)$.

In this work, given a structure x, we initialize the duplicate set with $\{z = f_\theta(x)\}$, and implement a sampling-based, Monte-Carlo-style *Token Duplicator* to expanding the duplicate set. We randomly mutate the residue-wise backbone tokens in z according to the similarity matrix of token embeddings, yielding a perturbed z'. We accept the proposed mutation z' with a probability proportional to the reconstruction quality of $g_\phi(z')$, based on which the next residue-wise mutation is performed. If $g_\phi(z')$ is sufficiently close to x (defined as TM-score(x, $g_\phi(z')$) > 0.9), we add it to the duplicate set.

During generative training of protein structures x via ProTokens z, we first resort to the *Token Duplicator* and construct a sufficiently large duplicate subset $\tilde{Z}(x)$. The L_1 or L_2 is then optimized based on $\tilde{Z}(x)$ in order to perform maximum likelihood estimation of x. This approach is similar to data (or label) augmentation which plays key role in many state-of-the-art generative AI models.

C. Encoder

1. Structure Encoder

Considering the separation of timescales, we encode the sidechain and backbone structures separately. As for the *backbone structure encoder* $f_{\theta_1}(x_{BB})$, an SE(3)-invariant Transformer based on invariant point attention(*19*), is designed to transform the Cartesian coordinates of the N, CA, C, O atoms of each residue to a SE(3)-invariant vector. The model is composed of an SE(3)-invariant HyperFormer(*20*) and a structure-aware Transformer. Specifically, the HyperFormer treats residues and inter-residue geometries as vertices and edges of a graph, respectively. Like EvoFormer in AlphaFold2, HyperFormer updates both the vertex and edge representations interdependently via hyper-attention mechanism. Based on the refined vertex (or single) and edge (or pair) representations output by HyperFormer, invariant point attention introduced in AlphaFold2 is adopted in the structure-aware Transformer, in order to efficiently learn the global geometric features (especially, long-range interactions) of a protein backbone.

On the other hand, since the equilibration of sidechain conformations is much faster than the backbone, all relaxed conformations of a sidechain (e.g., according to Boltzmann distribution) in the context of a given backbone can be considered as a single metastable state and reasonably embedded by a single vector which can distinguish the chemical identity of the sidechain fragment.

Therefore, we transact the amino acid embedding learned by AF2 as the s*idechain structure encoder* $f_{\theta_2}(x_{SC})$ without further optimization.

The embedding of an all-atom structure is obtained via the Cartesian product operation, that is, by concatenating the backbone embedding and sidechain embedding.

2. Deduplicator

Note that our choice of sidechain encoding is naturally invariant to perturbations of sidechain conformations. However, unlike the *sidechain structure encoder*, one particular concern of a learned *backbone structure encoder* is that the *robustness* of the yielded codes against subtle structure perturbation is not guaranteed. To alleviate this issue, we append a *Deduplicator* u_θ to the *backbone structure encoder* (fig. S13c), which is trained to surjectively map structures which belong to the same metastable states (or merely differ mutually up to negligible perturbations) to almost the same embedding,

$u_{\theta_1} \circ f_{\theta_1}(x_{BB}) \approx u_{\theta_1} \circ f_{\theta_1}(x_{BB} + \Delta x_{BB})\#(S3.11)$

where Δx_{BB} denotes the structural fluctuation within a metastable state. In terms of physics, the *Deduplicator* behaves like a "relaxation simulator" which relaxes fluctuated structures within a metastable ensemble towards a single stable representative structure, and consequently, degenerates the embeddings for fluctuated structures.

D. Tokenizer

The Tokenizer is a composition of a *Clustering* module s_θ (which has been elaborated at length in the main text), and a *Compressing* module r_θ.

1. Compressing Module

The ProToken of all-atom structure is obtained via $z = z_{BB} \otimes z_{SC}$, that is, the Cartesian product of the backbone token set and the sidechain token set. The continous ProToken embedding is equivalent to concatenating the the backbone embedding and sidechain embedding together, which has the shape of $(N_{res}, d = d_{BB} + d_{SC})$ for a N_{res}-long protein.

Note that without processing, d_{BB} and d_{SC} are usually large such that $d \gg 3\bar{N}_{atom}$ where $\bar{N}_{atom}$ stands for the average number of atoms per residue. For instance, in our model, $d_{BB} = 32$ during training and $d_{SC} = 256$ in consistency with AF2. Despite of being SE(3)-invariant, the dimensionality of such a representation is still much too higher than the intrinsic degrees of freedoms of a protein (which is upper bounded by the number of Cartesian coordinates of its structure).

Therefore, we include a *Compressing* module $r_\theta(v): R^{N_{res} * d} \to R^{Q * c}$ to concentrating the information of ProToken by lowering its dimensionality. The compression can be performed lengthwise and (or) depth-wise. As shown in fig. S13d, for the lengthwise compression, we transformed a ProToken string of shape (N_{res}, d) into (Q, d), with Q being a predefined number independent of and

usaually smaller than the average N_{res}. In this research, we performed the depth-wise compression, which reduces the shape of (N_{res},d) into $(N_{res},c \ll d)$, and we achieved this goal by means of dimensionality reduction methods(*21*).

Training of ProToken and PT-DiT

A. ProToken

Overall, the ProToken Distiller (g_ϕ, and $f_\theta \circ h_\theta$) is optimized towards the following coupled objectives:

i) minimizing the divergence between $p_\phi(x)$ and $p_D(x)$;

ii) maximizing the mutual information between ProTokens z and $g_\phi(\epsilon;z(x))$, while minimizing the mutual information between ProTokens z and the input structure x;

iii) minimizing the divergence between $f_\theta(x)$ and the encoding of the adversarial example $f_\theta(x')$.

Intuitively, the first objective ensures the *sufficiency* of ProTokens as a transformed representation of metastable protein structures. The second objective guarantees the *necessity* and *non-redundancy* of the transformed representation. The last objective improves the robustness of ProTokens against intrinsic structural fluctuations. Technically, these objectives can be achieved by minimizing an InfoGAN loss(*22*) regularized by variational information bottleneck(*3*) and adversarial training(*23*). As a result, the final loss function L_{PD} for the ProToken Distiller to be minimized is a linear combination of the sufficiency loss L_{suf}, necessity loss L_{nec}, and robustness loss L_{rob} with a reasonable set of hyperparameters,

$$L_{PD}(\theta,\phi) = L_{suf}(\theta,\phi) + L_{nec}(\theta,\phi) + L_{rob}(\theta)\#(S4.1)$$

1. Data preparation

In order to train the probabilistic Decoder, given each structure sample x_D from the training set, data augmentation is performed by means of metastable perturbation sampling, yielding a structure ensemble $\{x;x_D\}$ representing conformers from the same metastable state associated with x_D. Samples from $\{x;x_D\}$ are used to compute the generative loss defined in L_{suf}.

Furthermore, we construct adversarial examples $x^{'} \in \{x;x_D\}$ against x_D by setting a similarity cutoff TM-score$(x^{'},x_D) > 0.9$. These examples are provided to the model for the calculation of L_{rob}.

We note that both metastable conformers and adversarial examples can be prepared offline prior to the training, thus, incurring no extra overhead for training. Particularly, after the training proceeds, the adversarial examples can also be constructed online where the Decoder itself can

serve as a perturbative sampler of an input x_D. We will show that using these decoded structures as adversarial examples is indeed equivalent to the mutual information loss in L_{nec}.

2. Sufficiency

For the first objective, we adopt a loss function inspired by conditional GAN(*24*), which guides the *Decoder* to generate structures from the metastable states associate to an input structure x_D,

$$L_{GAN}(\theta,\phi) = -E_{x_D\sim D}\left[E_{z\sim p_\theta(z|x_D),\epsilon}D\left(g_\phi(\epsilon,z)||\{x;x_D\}\right)\right]\#(S4.2)$$

$$p_\theta(z|x_D) = h_\theta \circ f_\theta(x_D)$$

where $D(\cdot)$ is the critic measuring the divergence between two distributions; z is the token embedding (i.e., the identity of the metastable state) for x_D yielded by the Encoder and Tokenizer, and $p_\theta(z|x_D)$ depends on the code search algorithm during variational clustering (we adopt the nearest-code search(*14*) by default). ϵ represents random noises which are used to model the intrinsic structural fluctuations within the metastable state.

We repurpose the structure module of AF2 with the dropout trick(*25*) for $g_\phi(\epsilon,z)$, where ϵ denotes the random dropout mask, allowing the SE(3)-equivariant structure module of AF2 to generate different structures conditioned on the same token z. Noteworthy, other unconditional generative models for protein backbone structures(*1*) can also be adopted and conditionally fine-tuned.

To optimize this GAN objective, we implement maximum-mean discrepancy (MMD) as the critic $D(\cdot)$(*26*), a non-parametric integral divergence metric between two distributions, which is known to stabilize and simplify the training of GANs(*26*). The similarity kernel required by MMD is defined via frame aligned point error (FAPE)(*19*), a Fréchet-like distance metric(*27*) for protein 3D structures.

In order to differentiate through the Clustering module h_θ in Tokenizer, we approximate the gradient flow of backbone tokens via the straight-through (ST) estimator(*12*, *13*), and implement a commitment loss to reduce the errors of ST estimator,

$$L_{ST}(z_e,z_q) = (1-\beta_{ST})\|z_e - stop\_grad(z_q)\|_2^2 + \beta_{ST}\|z_q - stop\_grad(z_e)\|_2^2\#(S4.3)$$

where z_e, z_q represent vectors before and after quantization respectively. The final sufficiency loss for ProToken Distiller takes the following form,

$$L_{suf} = L_{GAN} + \lambda_1 L_{ST}\#(S4.4)$$

3. Necessity

We perform *variational clustering* in the Tokenizer according to a VIB objective. Specifically, the training objective of *ProToken Distiller* can be recast in terms of VIB theory(*3*),

$L_{VIB} = E_x[-log p_\phi(x|z) + KL[p_\theta(z|x)||p(z)]]\#(S4.5)$

In VIB, $p_\phi(x|z)$ is known as the prediction or reconstruction model (i.e., the Decoder), whereas $p_\theta(z|x)$ is the inference model (i.e., the Encoder). By appending a Tokenizer to the Encoder, the prior $p(z)$ and posterior $p_\theta(z|x)$ become discrete, the VIB in Eq. S4.5 simplifies to,

$L_{VIB} = E_x[-log\ p_\phi(x|z) + log\ K]\#(S4.6)$

where K is the vocabulary size. Therefore, to minimize L_{VIB} is equivalent to gradually pruning the backbone token vocabulary and minimizing K, which is achieved by re-clustering the embeddings into a smaller number of clusters during training(*28*).

Furthermore, to prevent the ignorance of conditional information (i.e., the ProTokens) by the generative *Decoder*, we also include a mutual information regularizer similar to InfoGAN(*22*), except that the auxiliary posterior estimator in InfoGAN is replaced by the conditional Encoder. This adaptation leads to a self-consistency term in the loss function,

$L_{MI}(\theta,\phi) =- E_{x_D \sim D}\left[E_{z\sim p_\theta(z|x_D),\tilde{x}\sim G(\epsilon,c)} log\ p_\theta(z|\tilde{x}) - H(p_\theta(z|\tilde{x}))\right]\#(S4.7)$

where the entropy term $H(p_\theta(z|\tilde{x}))$ is similar to the entropy regularization introduced in VQ training(*29*). The final necessity loss for ProToken Distiller takes the following form,

$L_{NEC} = \lambda_2 L_{MI}(\theta,\phi) + \log K\#(S4.8)$

4. Robustness

To help ProToken Distiller be better immune to adversarial attacks (i.e., structural fluctuations within a metastable state), we reuse the perturbative sampling data and present them as adversarial samples $x' \in \{x;x_D\}$ to the Encoder for each x_D, and apply an adversarial training loss for the Encoder,

$L_{ROB}(\theta) =- \lambda_3 E_{x_D\sim D,x'\sim\{x;x_D\}}\left[E_{z_D\sim p_\theta(z|x_D)} log\ p_\theta(z_D|x')\right]\#(S4.9)$

5. Training settings

ProToken Distiller is trained with a batch size of 288. The learning rate is set to 5e-4 with a cosine decay down to 2e-5 after 80,000 steps. The training is executed on 48 NVIDIA A100 GPUs.

The training is split into two stages. For the first 100,000 steps, we implemented the robustness loss L_{ROB} in Eq. S4.9, with a prepared set of adversarial examples but turned off the mutual information loss L_{MI} in Eq. S4.7. For the remaining 100,000 steps, we switched off the robustness loss, instead, MI loss was turned on.

6. Metastable Perturbation Sampling (MPS)

To sample more metastable conformations of a 3D structure x_0 associated with the same function, we performed metastable perturbation sampling according to the given 3D structure and yield $\{x\}$. Specifically, we recommend several options that can serve for MPS: 1) resorting to a MD simulation engine and running temporal proximal sampling(*30*); 2) resorting to AI-based sampler which can yield perturbed conformations like AF-Cluster(*25*); 3) self-distillation of a pre-trained probabilistic ProToken distiller. In this research, we implemented the first two strategies to obtain perturbed metastable conformations corresponding to a given reference 3D structure.

B. ProToken Diffusion Transformer (PT-DiT)

To prepare the training data of PT-DiT, we first performed Encoder inference for the training set of protein structures, yielding a basic set of ProTokens containing 551957 ProToken samples.

In order to counteract the biased estimation of likelihood of the latent diffusion model, we augmented the basic ProToken set with the duplicate set obtained by the *Token Duplicator*. Through experiments, we found empirically that augmentation of latent duplicates is vital for the success of training PT-DiT.

The generation quality of PT-DiT can be susceptible to errors that cause "token switch", so we introduced anisotropic diffusion kernel(*31*) for the variance-preserving diffusion process, in order to better align with the objective of the latent diffusion model

We trained PT-DiT with a batch size of 256, learning rate of 2e-4 for 1,000,000 steps, on 8 NVIDIA A100 GPUs.

Data Details

A. ProToken Training Dataset

Based on single-chain protein structures obtained from the RCSB Protein Data Bank (PDB) released before October 13, 2021, we performed data cleaning and filtering. We retained chains without structural gaps, excluded structures shorter than 30 residues, and omitted those derived from NMR experiments due to potential metastability issues. Ultimately, 551957 single-chain protein structures were used as training data. The list of PDB IDs and chain IDs is publicly accessible in the Open Science Framework (OSF) repository[16]. These PDB structures are accessible from the RCSB website (www.rcsb.org) using the corresponding PDB and chain IDs. Based on the training dataset, we further do data augmentation using the standard AlphaFold2 pipeline. The ground true structure is used as the template and no MSA is used. Dropout is used in the whole inference process and thus the result structure is more or less different from the original structure. we use this structure as the perturbed structure for further training and BLOSUM counting.

B. ProToken Reconstruction Validation Dataset

1. RCSB single chain dataset

We used the dataset curated by the PSP dataset(*32*) for validation of single chain reconstruction task. It includes two main parts: the CASP14 dataset and a new validation dataset. The new dataset was curated from two source of public data. One is CAMEO targets from October 16, 2021, to February 12, 2022, and another is new single clusters from the PDB with 40% identity between October 13, 2021, and March 15, 2022.

Thus, the validation dataset is unique and diverse compared to the training set. We filtered out samples shorter than 1536 residues for easier validation. In total, we have 513 non-overlapping samples. All ground truth structures were released after October 13, 2021, which is after all the training samples. This prevents any data leak during validation and testing. The validation dataset is provided in the OSF data repository.

2. CASP14 single chain dataset

To clearly measure the performance of ProToken Distiller on the widely recognized CASP14 dataset, we included all 87 single-domain regular targets with ground truth structures in CASP14 to form this dataset, even though this dataset is part of the earlier validation set. The CASP14 single-domain structures are available in the OSF repository[16].

3. CASP15 single chain dataset

All regular targets in CASP15 with ground truth structures are used to form this dataset, which results in 45 single domain protein structures. the CASP15 single domain dataset is available in the OSF repository[16].

4. AFDB dark cluster dataset

Foldseek has identified 711,705 dark clusters, which are likely enriched with novel structures(*33*). To ensure structural quality, we followed Foldseek data processing flow and selected 33,842 clusters with the highest average AlphaFold2 prediction confidence (average plDDT >90). From each cluster, we chose the member with the highest confidence for further investigation, similar to Foldseek. The structures in this dark cluster dataset can be downloaded from the AlphaFoldDB website (https://alphafold.ebi.ac.uk/), and the name list is available at (https://afdb-cluster.steineggerlab.workers.dev/).

5. CASP14 and CASP15 multi-domain dataset

CASP14 and CASP15 have provided "Domain Definitions" on their official website, which are used for multi-domain structure prediction tasks. Following the curation approach of DeepAssembly(*34*), we formed a total of 30 multi-domain targets: 17 from CASP14 and 13 from CASP15. These targets were used to evaluate the reconstruction ability for multi-domain protein structures. The 30 multi-domain structures are available at the OSF repository[16].

6. Multimer dataset

Datasets for the test of multi-chain protein reconstruction task were curated from AF2Complex benchmark sets(*35*), specifically Dimer1193 and Oligomer562. Since no multimer structures were

used during the training of ProToken Distiller, all multimer cases in these two sets were reserved for benchmarking the multimer reconstruction task.

7. PDBFlex conformation dataset

The PDBFlex database has organized structure clusters with similar sequences but significant structural differences(*36*). To assess ProToken's ability to distinguish and reconstruct various metastable states, we followed the database's definitions of Local RMSD and categorized the clusters into bins based on Local RMSD ranges of 2-4Å, 4-8Å, 8-16Å, 16-32Å, and 32-64Å. From each bin, we selected 10 clusters, totaling 50 structure clusters. From each cluster, we chose the pair of structures with the highest backbone RMSD, creating a final dataset of 100 structures. This dataset serves as the test set for the multi-conformation reconstruction task and all structures are available at the OSF repository[16].

8. APObind Pocket-Ligand Dataset

Based on the APObind ligand unbound protein conformations(*37*), we curated 229 *apo* structures aligned with their corresponding *holo* protein-ligand structures. In total, 458 proteins were used for the pocket reconstruction test. The pocket is defined according to the AF3 methodology, encompassing all heavy atoms within 10 Å of any heavy atom of the ligand. The backbone of a residue includes the 'N', 'C', 'CA', and 'O' atoms. The pocket backbone RMSD was calculated after aligning all backbone atoms of the *apo-holo* conformer pairs using Biopython and Numpy in Python scripts. The pocket residue indexes, *apo-holo* protein structure pairs together with the ligand structures are available at the OSF repository[16].

9. Antibody CDR dataset

Following the DeepAb test set(*38*), we used 92 antibody cases from the RosettaAntibody benchmark set (47 targets) and a set of clinical-stage therapeutic antibodies (45 targets) to form the antibody CDR dataset. We cleaned and annotated these protein complexes, resulting in a total of 238 single-chain structures. The Chothia CDR loop definitions were used to measure RMSD throughout this work. All the single-chain structures, FASTAs and annotations are available at the OSF repository[16].

C. PT-DiT Training Dataset

We randomly utilized 550957 ProToken sequences of the structures and corresponding amino acid sequences from the ProToken Distiller training set for PT-DiT training and the rest 1,000 data points serve as the validation set. The name lists for both the training and validation sets are available at the OSF repository[16].

<u>Additional validation experiments of ProToken</u>

A. ProTokens generalize to the dark protein universe, disconnected domain assemblies and multimers

The test set in the above experiment consists exclusively of structures from experiments, which may be subjected to human biases. Because ProTokens are trained solely on experimental structures, it is possible that such biases are inherited. Based on an AI-predicted protein structure dataset (AFDB)(*39*), previous research reveals that there is a large volume of unexplored dark space of the protein universe(*40*). Therefore, we extract high-confidence structures from the AFDB dark cluster dataset and interrogate ProTokens' capability of reconstructing these out-of-distribution (OOD) folds. Fig. S13a shows that these OOD single-chain structures can be represented by ProTokens as well, and the reconstruction quality shows no significant difference from that of the experimental targets statistically. This experiment demonstrates that ProTokens are generalizable for tertiary structural patterns.

Next, we wonder whether ProTokens are able to capture quaternary structural patterns, despite the fact that no such examples were presented during training. Considering that multi-domain proteins are often regarded as transitioning from tertiary to quaternary structures, we first collect several samples containing multi-domain chains from CASP14 and CASP15 test sets. These single chains are manually chopped into annotated domains according to the official definition, yielding a set of disconnected multi-domain assemblies which resemble protein complexes. Noteworthy, the training set of ProTokens merely contains single-chain structures without discontinuity (i.e., without structural gaps), so these multi-domain assemblies are also OOD examples. Nevertheless, we allow ProTokens to treat them by manually setting a residue index gap between domains during inference. We surprisingly find that ProTokens can reconstruct the assembled structures reasonably well (fig. S2d) when the inter-domain contacts are not sparse.

Based on this encouraging result, we further test ProTokens over real-world protein complexes, including homomers and heteromers. The overall performance is satisfying in that both the single-chain and the complex structure can be well reconstructed (fig. S2e). This result confirms ProTokens' capability of characterizing both the tertiary and quaternary structure patterns, although slight performance degradation is observed when the protein complex involves more chains (fig. S2e).

In addition to the overall shape, the accuracy of the complex interface is of particular interest when assessing a multimer structure. Motivated by this, we compute the DockQ score(*41*) for the tested dimers, including antigen-antibody (Ag-Ab) complexes(*35*). Fig. S2f shows that the interface is relatively accurate(*41*), though not perfect, with an average and median DockQ exceeding 0.49. Noteworthy, compared to multi-domain assemblies or heterodimers, we find that the reconstructed Ag-Ab complex interface shows larger variance, although each single chain is reconstructed well (fig. S2g). This may be due to the fact that the interaction patterns between Ag-Ab are often sparse and largely determined by the side chains, hence, are less comparable to the tertiary structural patterns that ProTokens are trained on. Besides, the flexibility of the Ag-Ab interface may also impact the fidelity of ProTokens.

Nevertheless, these experiments on multi-chain structures validate the generalization of ProTokens, which are trained solely on tertiary structures, over quaternary interactions. Our observation also implies that the quaternary structure patterns between protein chains may share deep connections with tertiary patterns within a chain, both arising from fundamental physics interactions between amino acid residues.

B. ProTokens are descriptive of finer functional protein conformations

Researchers are particularly concerned with alternative conformations that may lead to functional switching of a protein. Despite many efforts being paid(*25*, *42–44*), decoding alternative protein conformations according to the amino acid sequence remains an unresolved challenge. According to the probabilistic tokenization theory, such difficulty is largely due to the compactness of Anfinsen's tokens (fig. S12), leading to large and multimodal intra-state variation which is hard to model. Compared to the Anfinsen's tokens, ProTokens are designed to be more informative by compromising its compactness. In principle, different metastable conformational ensembles of one protein, which are degenerate in terms of Anfinsen's tokens, can now be distinguished by ProTokens.

Many proteins are known to undergo conformational changes or adaptations during binding to ligand(s), we thus wonder whether ProTokens can characterize the binding-altered backbone conformations of a protein. Similar to the previous experiment (see Results), we test the reconstruction quality of a set of proteins with different *apo*- and *holo*-form conformations (defined as conformers mutually different with a minimum backbone RMSD larger than 2.0 Å with and without ligand binding). According to the results shown in Fig. 4f, ProTokens are able to faithfully describe both the *apo* and *holo* conformers of a ligand-binding protein and preserve their relative differences. This important feature permits the use of ProTokens for the design of ligand-binding proteins involving conformational changes.

C. Fidelity of ProTokens reflects the (meta)stability of 3D structures

As observed in the previous section, the fidelity of ProTokens seem to correlate with the stability of a protein structure, possibly due to the probabilistic tokenization process, that is, the metastable structure patterns are prioritized by ProTokens over the transient and dynamic snapshots of a protein 3D structure. To further investigate this phenomenon, we zoom in our analysis over protein residues, considering that even within a single protein, the stability of different residues may be heterogeneity. For instance, it is common that some substructures are intrinsically more dynamic or less stable than the other regions, which may result in less confident or even unresolved parts in experimentally determined structures.

Therefore, we disassemble the test samples into residues, and categorize them according to the B-factors reported by the crystallization experiments. In general, a larger B factor indicates larger

intrinsic dynamics (or less stability) of the residue in its 3D structure(*45*). Not surprisingly, we find that the fidelity of a ProToken corresponding to a certain residue correlates with the B-factor of that residue (fig. S9a). In other words, if the local structure is less stable, it is also more likely to be smeared by ProTokens. We further corroborate this conclusion based on another test set consisting of AF2-predicted structures. Previous research revealed that the predicted confidence (predicted lDDT) of AF2 can reflect to a certain degree the structural flexibility of the residue(*46*). In line with previous findings, we observe that on average, when the pLDDT of a residue is lower, the fidelity of ProTokens for that residue gets worse (fig. S9b).

Additionally, as many loop or intrinsic disordered regions of the protein usually cannot be resolved by crystallization due to lack of metastability, it is likely that ProTokens are not able to represent them either. To test this hypothesis, we manually fix and fill the unresolved regions of the experimentally determined structures using PDB-Fixer(*47*) and AF2, then compute the reconstruction quality of ProTokens for these regions. Consistent with the previous conclusion, we observe a significant drop in the fidelity of ProTokens for these disordered regions (fig. S9c). However, we notice that, unlike the manually fixed random regions, the experimentally resolved loop conformation of an antigen-bound antibody can be well reconstructed from ProTokens. This observation implies that the antigen-binding loop of an antibody is a metastable conformer of a free-form antibody, in line with the thermodynamics theory of protein binding. Unlike intrinsically disordered loops, the difficulty in experimentally resolving these metastable loop conformers alone may result from the existence of other competing metastable conformations.

Additional validation experiments of PT-DiT

A. PT-DiT shows emerging capability for inverse folding

Inverse folding is an important task in structure-based protein design when the sidechain and backbone structures are designed in a factorized way, and many efforts have been paid to develop powerful inverse folding models. Noteworthy, all of these models underwent supervised training towards the specific inverse folding objective, regardless of whether they make use of other pretrained models.

Due to the unified modality of 1D and 3D structures in ProTokens, the inverse folding task can be translated as in-filling or generating part of the ProTokens (i.e., sidechain tokens) given the remaining part of ProTokens (i.e., backbone tokens). Intriguingly, in the framework of PT-DiT, this definition coincides with the well-known inpainting task in image generation. Therefore, we can directly transact useful tools and techniques developed for image inpainting to the inverse folding task. Particularly, it is known that image inpainting can be achieved in a zero-shot manner based on a well-trained diffusion model. We thus implement a simple and useful zero-shot inpainting technique which allows PT-DiT to conduct inverse folding tasks without any fine-tuning. Specifically, we first encode a given backbone structure into backbone token embeddings,

then guide the PT-DiT to generate the remaining embeddings (i.e., the sidechain tokens), and obtain the sidechain tokens corresponding to the amino acid sequence.

In Fig. 3e, we showcase that given a backbone structure, even without fine tuning, PT-DiT can generate side chains with relatively high sequence recovery with respect to a known all-atoms structure. However, the recovery is not an ideal measure for inverse folding considering that many different amino acid sequences may fold into nearly the same backbone structures. Since PT-DiT is a generative model, we can sample different sidechain tokens given the same backbone structure, and yield diverse inversely folded sequences. We find that many of the generated sequences, despite being less identical to the reference sequence, are predicted to fold into the designated backbone structure according to a structure predictor (fig. S10). From these results we can conclude that PT-DiT shows emerging capability for probabilistic inverse folding task, that is, being able to generate conservative as well as diverse amino acid sequences compatible with a designated (foldable) backbone structure without any fine-tuning.

B. PT-DiT enables flexible protein engineering through contextual design

In addition to de novo design, many researchers are interested in contextual design, for instance, design structures conditioned on part of known structures or sequences. Similar to inverse folding, in the framework of PT-DiT many contextual design tasks are all equivalent to inpainting, hence, may be accomplished by a pretrained PT-DiT without fine tuning. We consider several contextual design scenarios that may find broad applications, and interrogate PT-DiT's capability of dealing with these challenging problems.

In the first scenario, PT-DiT is invoked to design a scaffold that can accommodate a specified binding pocket of a ligand (e.g., a small molecule), given the all-atom coordinates of the binding pocket as the context. This scenario is particularly relevant to designing ligand-binding proteins, which remains difficult to both sequence-based and structure-based protein design methods.

In fig. S3, we showcase designed proteins for two small molecule ligands. In each case, the pocket is obtained by cropping the structure of a ligand-binding protein and only keeping the binding pocket in a continuous form (that is, no gap between residue indices), mimicking a pre-designed binding site. ProTokens (both backbone and sidechain tokens) for this binding pocket are first obtained via the Encoder, and the length of the flanking tokens to the pocket is randomly sampled. The remaining task is to infill the ProToken embeddings for the added flanking blanks. After inpainting is done, we first detokenize the infilled ProTokens into 3D structures via the Decoder. It can be seen that the shape of the binding pocket is preserved in the decoded structure, with a designed flanking sequence as the scaffold. We also validate the generated all-atom structure through a structure predictor, and confirm that the predicted structure aligns well with the generated structure, further demonstrating the potentiality of PT-DiT as a zero-shot designer for ligand binding proteins.

C. Directed Evolution in the Latent Space of PT-DiT via Active Learning

Our active learning algorithms closely resemble those used in EVOLVEpro, with key settings, including the strategy for selecting first-round mutants (random), the top-layer regression model (random forest), and the active learning policy for selecting next-round variants (top-n), being identical to those in EVOLVEpro. The only difference is the input for the regressor. In EVOLVEpro, the residue-wise embedding from ESM-2 is averaged along the residue dimension to generate a protein embedding (dim=5120). We adopt a similar strategy for PT-DiT embeddings (dim=40), but we found that incorporating the second moment improves performance, resulting in a total embedding of (dim=80) that integrates both sequence and structural information. The structures used as the PT-DiT input are predicted by AlphaFold3 online server according to the input sequence, and the residues of N-ter and C-ter with pLDDT below 0.7 are ignored.

Fig. 5g–i and Fig. S7 demonstrate that the embeddings from PT-DiT and ESM-2 individually yield comparable results across the 12 deep mutational scanning datasets, with neither consistently outperforming the other. Notably, combining the two embeddings tends to produce the most robust and reliable performance. However, PT-DiT performs poorly on the MAPK1 kinase and TEM-1 beta-lactamase datasets. Since PT-DiT's embeddings are learned in a fully data-driven and black-box manner, they are currently difficult to interpret, and we are unable to offer definitive mechanistic explanations for the underperformance. Nevertheless, we believe this limitation could arise from the following two reasons,

1) **Bias in the structure used for inference:** As detailed in the "Directed Evolution in the Latent Space of PT-DiT via Active Learning" section of the Supplementary Text, the structural input for PT-DiT inference was obtained from the AlphaFold3 server rather than experimentally resolved crystal structures. These predicted models may contain noise or biases. Furthermore, MAPK1 is a kinase that adopts distinct conformations in its apo and holo states, particularly in the activation loop and substrate recognition regions—conformational changes that are often functionally relevant. Similarly, TEM-1 beta-lactamase, as an enzyme involved in catalyzing biochemical reactions, also undergoes conformational shifts upon substrate binding and product releasing. Therefore, relying on a single static structure may fail to capture the relevant functional states, limiting the model's ability to accurately predict the effects of mutations.
2) **Sensitivity of enzyme's side chain structures in catalytic area:** The function of enzymes often depends critically on atomic-level interactions between the protein and its substrate, meaning that the precise conformations of side chains in the catalytic region are essential for accurately predicting activity. However, the structural information used in PT-DiT inference is limited to backbone representations, which may reduce the model's effectiveness in capturing these subtle but functionally important features. Additionally, in terms of sequence embeddings, PT-DiT has significantly fewer parameters compared to ESM-2, and is pretrained on a smaller dataset, given the relative scarcity of structural data compared to sequence data. These factors may contribute to the reduced performance of PT-DiT in tasks requiring high-resolution functional insight.

Calculation of TM4-TM6 distance of μ-Opioid Receptor

For the DEER experiment on the μ-opioid receptor, we sample the possible conformations of the small molecule HO-1427 used in the experiment using mtsslWizard, and calculate the distribution of distances between fluorescent molecules and the average distance.

Time-Lagged Independent Component Analysis and Clustering of MD Simulations

We performed dimensionality reduction analysis on the Abl trajectory (see method) using TICA. The implementation of TICA was based on the Python package pyemma, with lag time set to10.

Fig. S1.

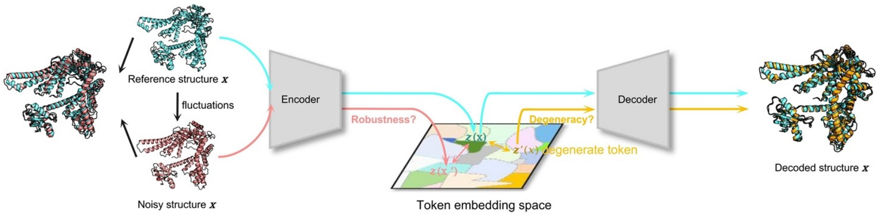

Pitfalls in tokenization of protein 3D structures. Machine-learned tokens may suffer from non-robustness (red) and degeneracy (yellow).

Fig. S2.

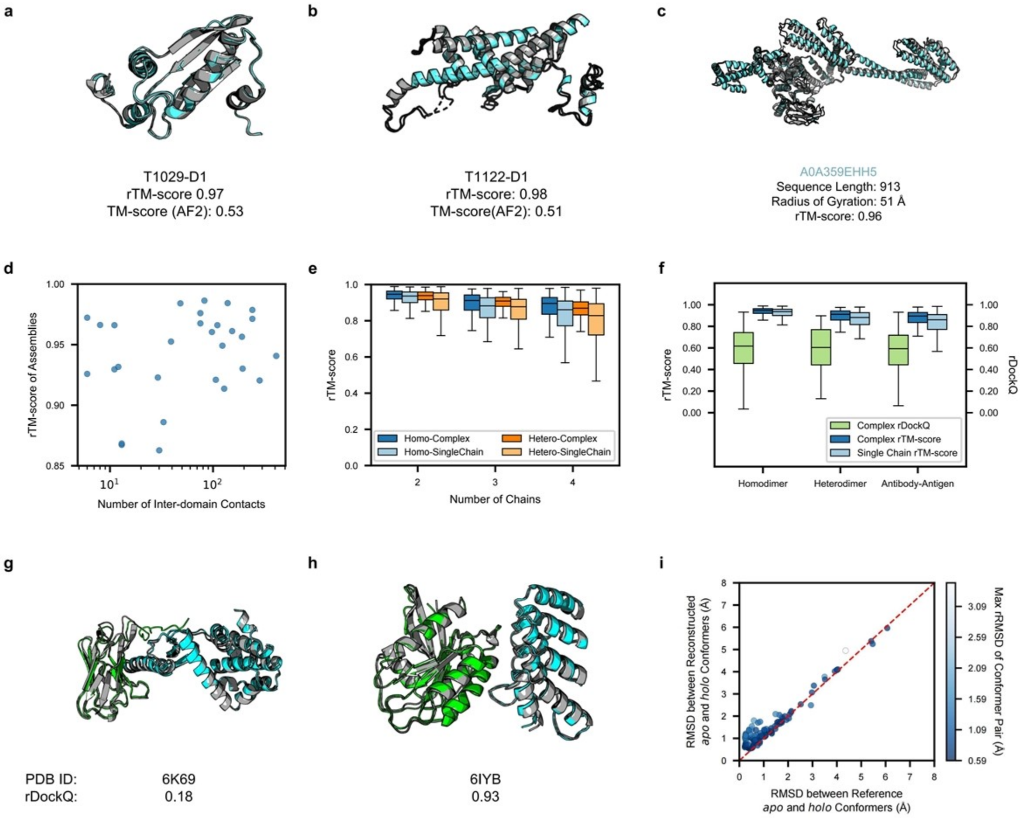

ProToken reconstruction performance on test datasets. a, Reconstructed structure of T1029-D1 (cyan) from CASP14 test dataset aligned with the ground truth structure (gray). The rTM-score of ProToken is 0.97 while structure predicted by AlphaFold2 is 0.53. **b,** Reconstructed structure of T1122-D1 (cyan) from CASP15 test dataset aligned with the ground truth structure (gray). The rTM-score of ProToken is 0.98 while structure predicted by AlphaFold2 is 0.51. **c,** Reconstructed structure showcases ProToken's performance on long sequence. **d**, ProTokens can reconstruct in high quality the disconnected domain assemblies with sufficient inter-domain interactions. The sample size is 28 in total. The inter-domain contact is defined as any CA-CA atom pair that distance is below 8 Å. **e**, ProTokens characterize both the tertiary and quaternary structure patterns in protein complexes. **f**, The interface between homo- and hetero-dimers can be reasonably reproduced by ProToken. **g-h**, Compared to heterodimer (PDB ID: 6IYB), degraded fidelity is observed for the antigen-antibody interface (PDB ID: 6K69) with sparser quaternary interactions. **i**, Assessing ProTokens' capability of distinguishing apo and holo conformers for ligand-binding proteins. The box plots in b and c are defined by the median as the center black line, first and third quartiles as the box edges and 1.5 times the interquartile range as the whiskers

Fig. S3.

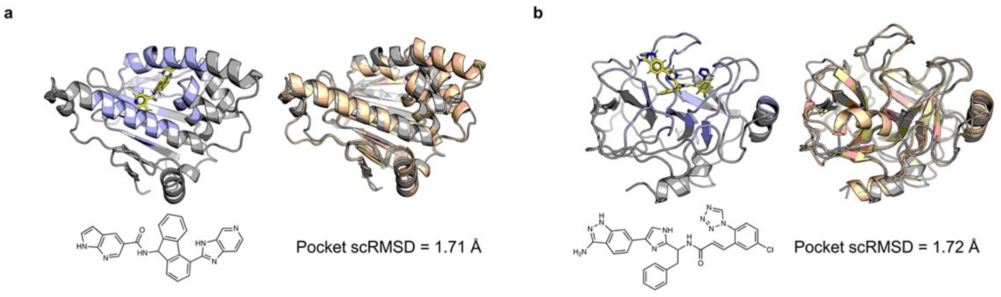

PT-DiT enables zero-shot contextual design of ligand binding proteins. a-b, Design of scaffold (gray) for ligand (yellow) binding proteins based on pocket structures (blue). The predicted structure (yellow) given the designed sequence is superimposed with the designed folds (gray), showing preservation of the desired pocket structure. Ligands are shown at bottom.

Fig. S4.

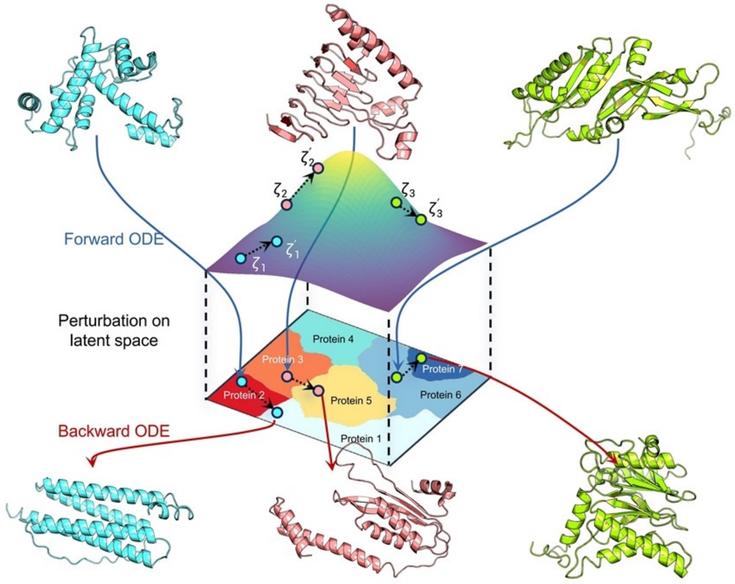

Illustration of controlled evolution over the PT-DiT latent space. Three proteins (colored in cyan, red and green, respectively) are evolved into new folds by perturbative extrapolation of their embeddings over the PT-DiT latent space.

Fig. S5.

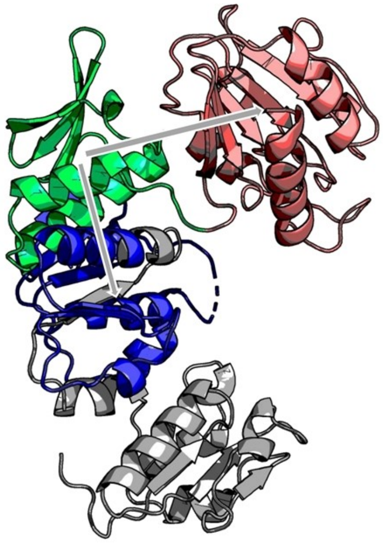

Showcase of MurD structure and the collective variable. The collective variable is defined as the angle between the center of mass of three selections: residues 120-230 (blue) and 230-299 (green) and 299-437 (red) for domain 1, 2, and 3, respectively. The vectors from green domain to red domain and from green domain to blue domain are shown with two gray arrows. The PDB ID of the structure is 1E0D.

Fig. S6.

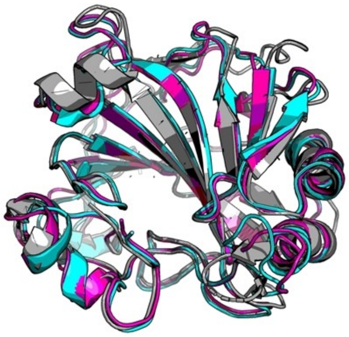

Validation of de novo designed Carbon anhydrase sequence. The generated sequence's structure predicted by AlphaFold3 (cyan) and ESMFold (red) is aligned with the ground truth structure (PDB ID: 3JXG).

Fig. S7.

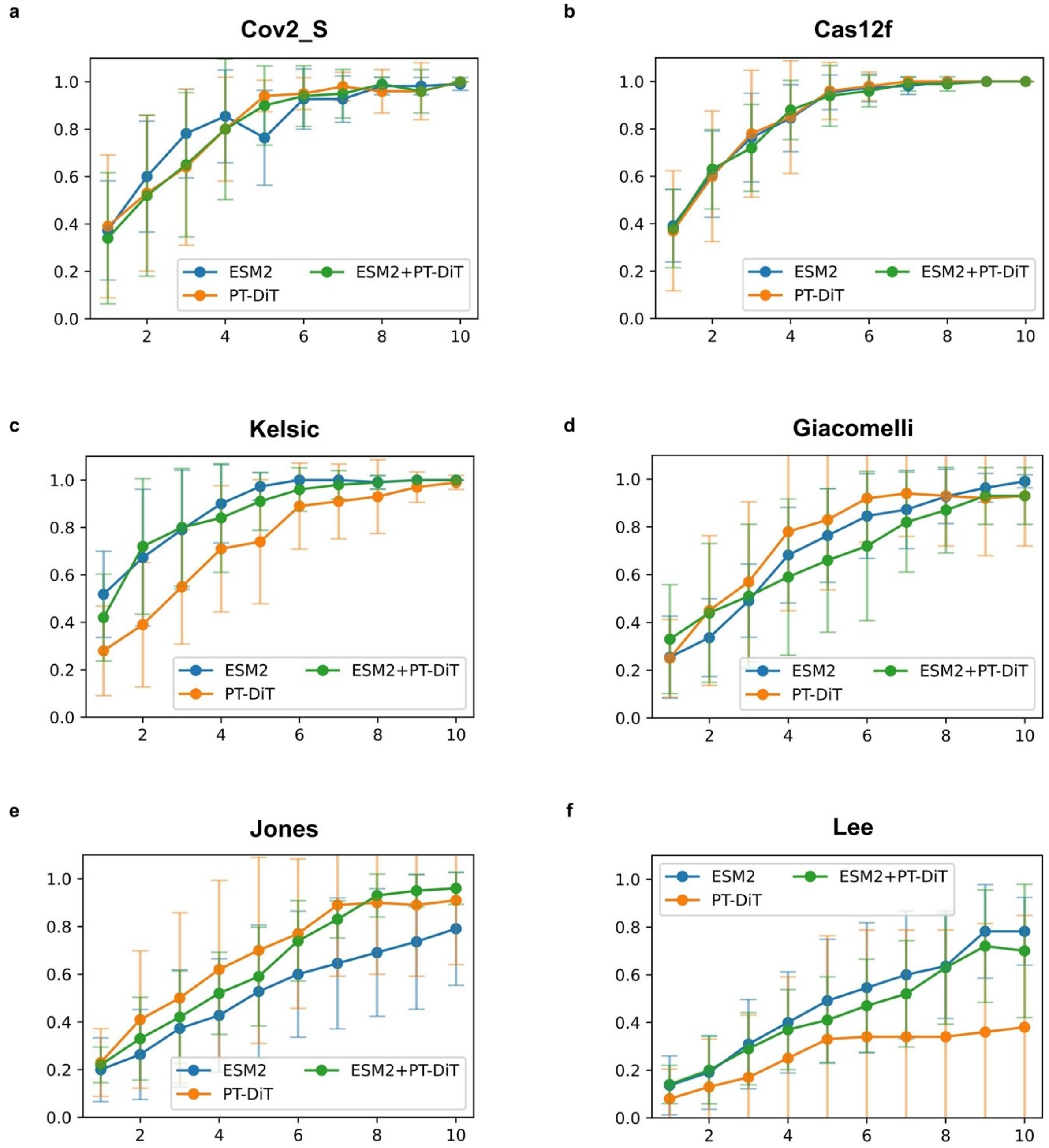

Directed evolution performance of PT-DiT compared with EVOLVEpro. The high activity candidate percentage of 10 rounds simulated directed evolution across different DMS datasets.

Fig. S8.

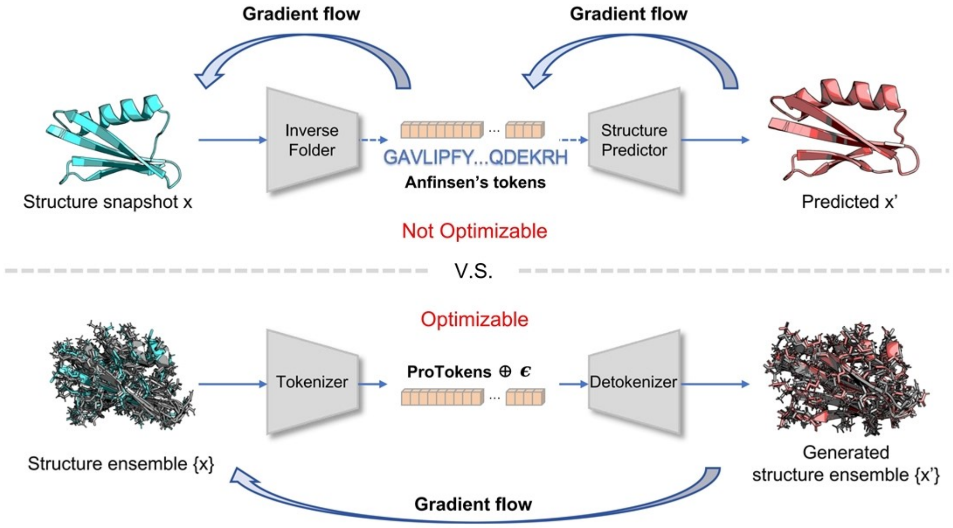

ProToken combines traditional structure prediction and inverse folding tasks. ProTokens can be learned by connecting inverse folding and structure prediction models end-to-end, and replacing amino acids by optimizable vocabulary.

Fig. S9.

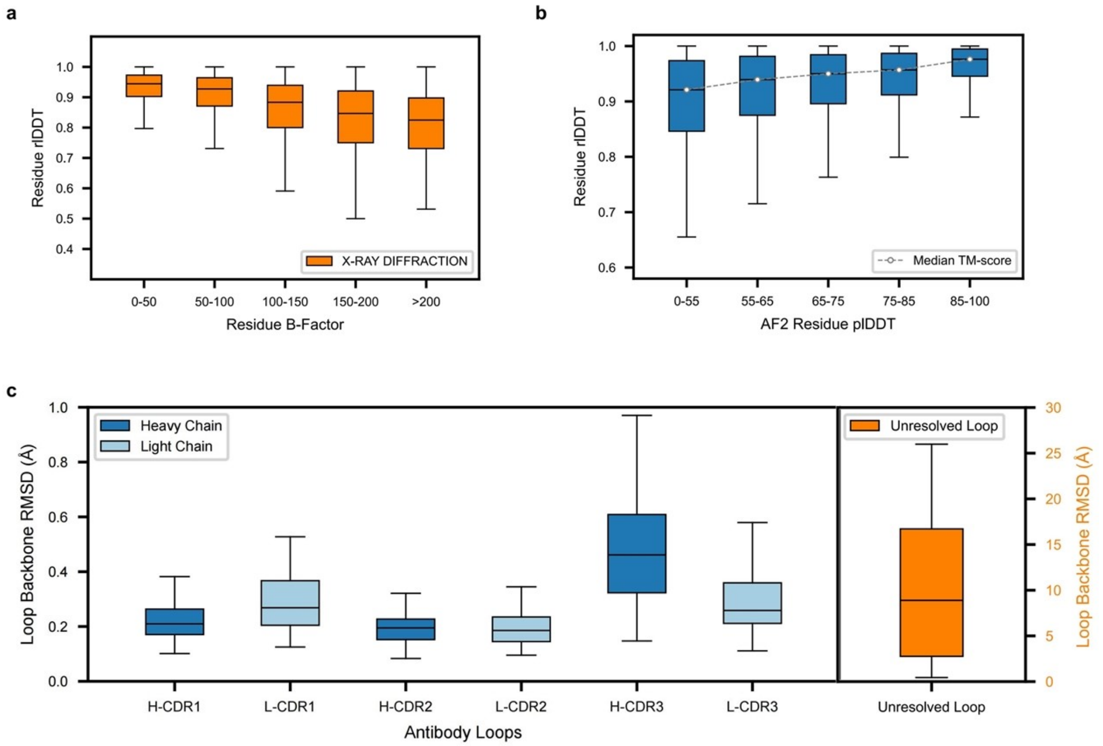

ProTokens reflect similarity and metastability of protein structures. a, The fidelity of ProTokens correlates negatively with the flexibility of local structures as measured by experimental B factors. b, The fidelity of ProTokens correlates positively with the stability of local structures as indicated by AlphaFold2 pLDDT values. c, Antigen-binding conformations of antibody loops are represented well by ProTokens (left panel), indicating metastability, in contrast to experimentally unresolved loops (right panel; note the scale of y-axis is different from the left panel).

Fig. S10.

a

b

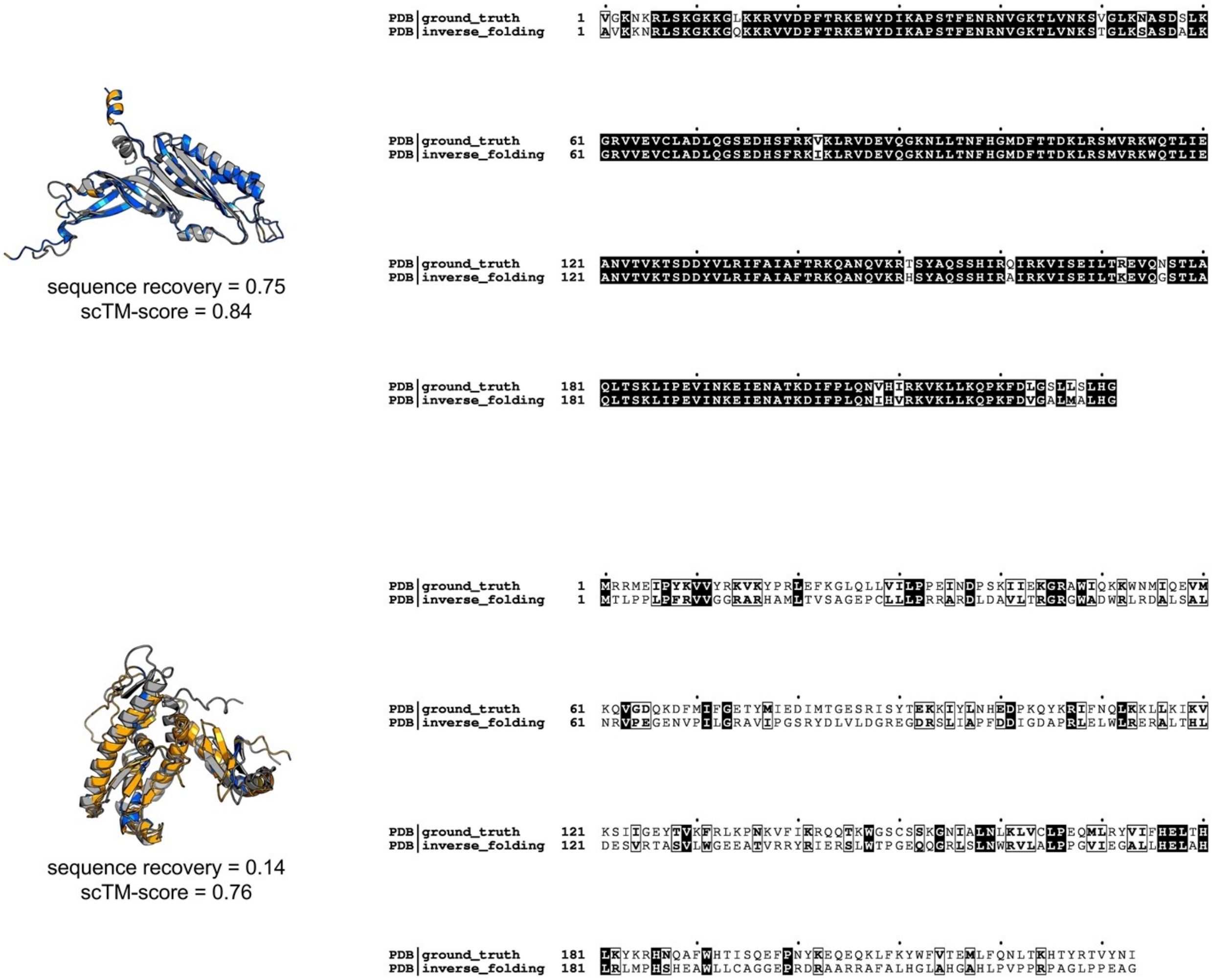

Case Study of Inverse Folding Tasks empowered with PT-DiT. The ground truth structure (gray) and structure predicted by ESMFold based on the generated sequence of PT-DiT are aligned and shown on the left. The common residues are colored in marine and uncommon residues in orange. The ground truth sequence and sequence generated by PT-DiT are aligned on the right. The common residues are colored in black background. The upper case has a high sequence recovery and high self-consistency TM-score of 0.84, while the lower case has a low sequence recovery, while maintain relatively high self-consistency TM-score of 0.76.

Fig. S11.

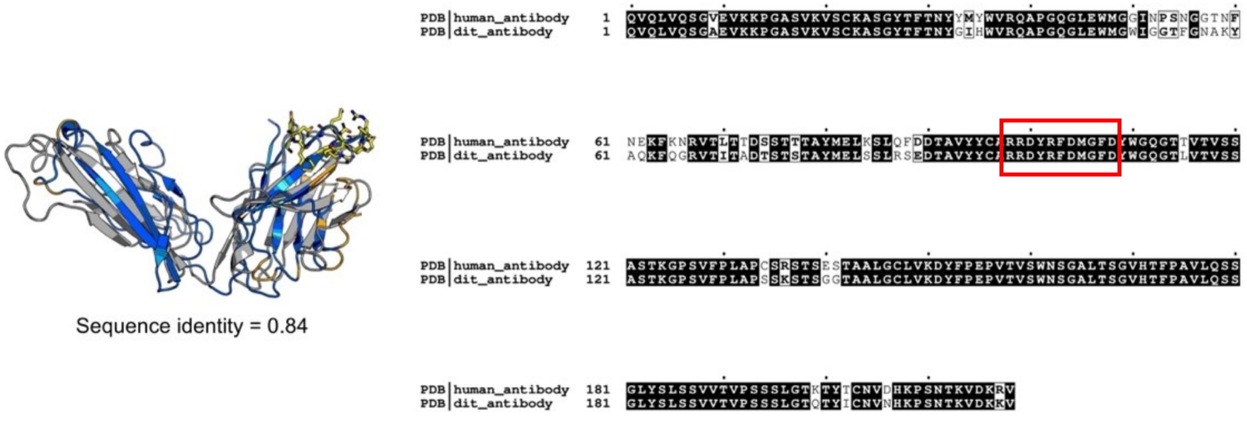

Comparison of sequence and structure between PT-DiT generated antibody candidates and a human antibody. The sequence generated by PT-DiT compared to the human antibody sequence. The CDR3 residues are marked in red square and the common residues in black background. Sequence identity of two structures is 0.84.

Fig. S12.

Probabilistic tokenization of protein 3D structures. a, Metastable states defined at different timescales can lead to different tokens of protein structures, including amino acids.

Fig. S13.

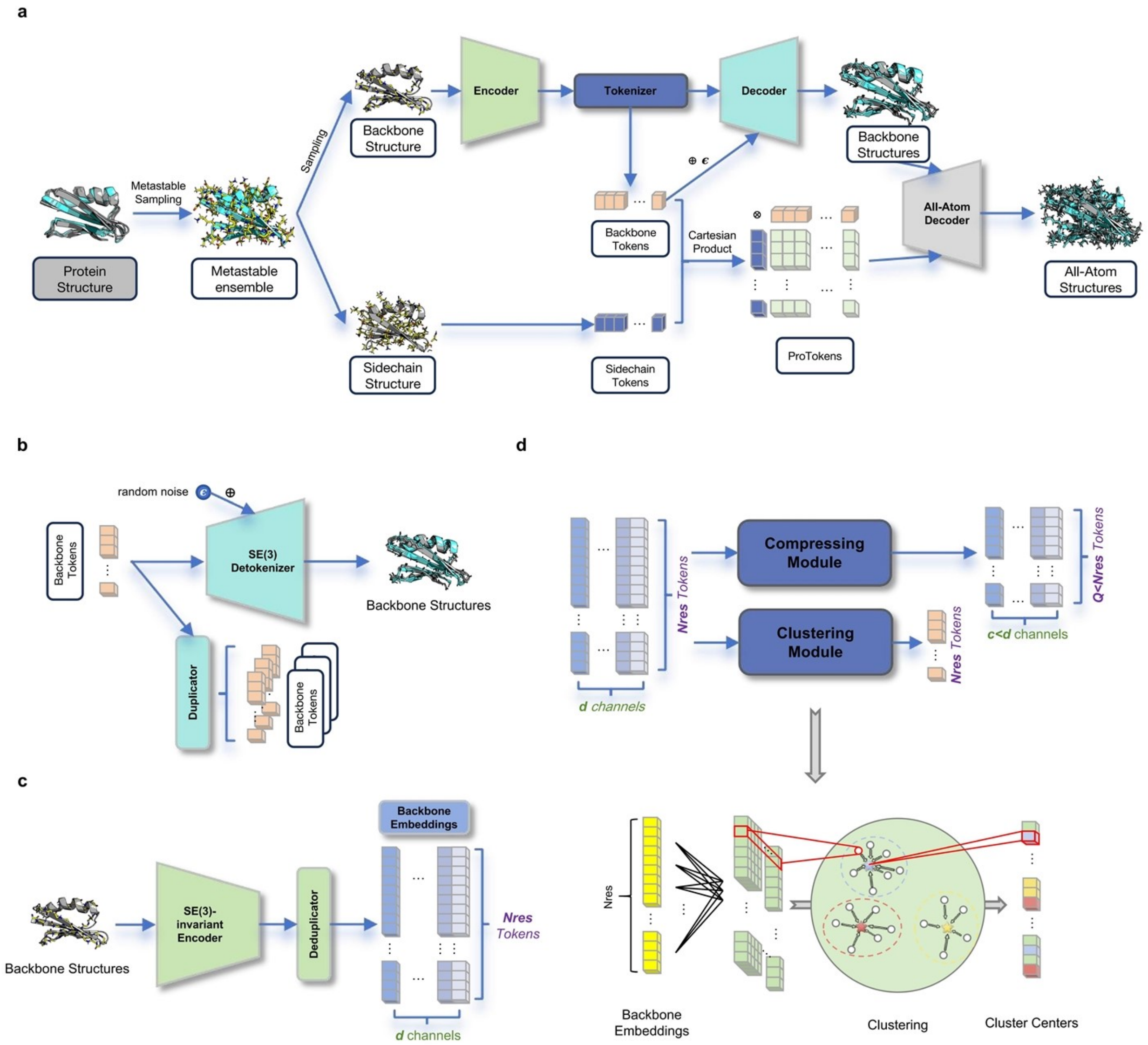

Architecture of Protein tokenization module. a, Overview of the protein tokenization module. The all-atom structure(s) is tokenized through the backbone track and sidechain track separately, leading to backbone tokens and side chain tokens, respectively. ProToken is the Cartesian product of the two. Protein tokenization module consists of a generative Decoder (**b**), an Encoder (**c**), and a Tokenizer (**d**).

Table S1.
Proteins in experimental datasets[a]

Type[b]	Sample Name	PDB Entry	Description	Reference
Metastable state sampling	Abl_active	1OPJ	The active state of Abl	David E. Shaw et. al.(*48*)
	Abl_inactive	2F4J	The inactive state of Abl	David E. Shaw et. al.(*48*)
	MurD_open	3UAG	The open state of MurD	Matteo T. Degiacomi(*49*)
	MurD_close	1E0D	The close state of MurD	Matteo T. Degiacomi(*49*)
	MurD_inter	5A5E	The intermediate state of MurD	Roman Šink et. al.(*50*)
	μOR_active	6DDF	The active state of μ-opioid receptor	Chunlai Chen et. al.(*51*)
	μOR_inactive	4DKL	The inactive state of μ-opioid receptor	Chunlai Chen et. al.(*51*)
Evolution-like protein discovery	SUMO	1U4A	Human SUMO-3 C47S	Yunyu Shi et. al.(*52*)
	Ubiquitin	1D3Z	Human Ubiquitin structure	Gabriel Cornilescu et. al.(*53*)
	Carbon anhydrase_1	3DCW	Human carbonic anhydrase IX	Robert McKenna et. al.(*54*)
	Carbon anhydrase_2	3JXG	CA-like domain of mouse PTPRG	Samuel Bouyain et. al.(*55*)

a. This table includes details of all the experimental resolved structures, and details of 12 DMS dataset can be found in the supplementary information Table S2 of Omar O. Abudayyeh et. al.
b. Type means the applications of PT-DiT corresponding to the contents in the results of main text.

Reference

1. J. L. Watson, D. Juergens, N. R. Bennett, B. L. Trippe, J. Yim, H. E. Eisenach, W. Ahern, A. J. Borst, R. J. Ragotte, L. F. Milles, B. I. M. Wicky, N. Hanikel, S. J. Pellock, A. Courbet, W. Sheffler, J. Wang, P. Venkatesh, I. Sappington, S. V. Torres, A. Lauko, V. De Bortoli, E. Mathieu, S. Ovchinnikov, R. Barzilay, T. S. Jaakkola, F. DiMaio, M. Baek, D. Baker, De novo design of protein structure and function with RFdiffusion. *Nature* **620**, 1089–1100 (2023).

2. J. Abramson, J. Adler, J. Dunger, R. Evans, T. Green, A. Pritzel, O. Ronneberger, L. Willmore, A. J. Ballard, J. Bambrick, S. W. Bodenstein, D. A. Evans, C.-C. Hung, M. O'Neill, D. Reiman, K. Tunyasuvunakool, Z. Wu, A. Žemgulytė, E. Arvaniti, C. Beattie, O. Bertolli, A. Bridgland, A. Cherepanov, M. Congreve, A. I. Cowen-Rivers, A. Cowie, M. Figurnov, F. B. Fuchs, H. Gladman, R. Jain, Y. A. Khan, C. M. R. Low, K. Perlin, A. Potapenko, P. Savy, S. Singh, A. Stecula, A. Thillaisundaram, C. Tong, S. Yakneen, E. D. Zhong, M. Zielinski, A. Žídek, V. Bapst, P. Kohli, M. Jaderberg, D. Hassabis, J. M. Jumper, Accurate structure prediction of biomolecular interactions with AlphaFold 3. *Nature* **630**, 493–500 (2024).

3. A. A. Alemi, I. Fischer, J. V. Dillon, K. Murphy, Deep Variational Information Bottleneck. arXiv arXiv:1612.00410 [Preprint] (2019). https://doi.org/10.48550/arXiv.1612.00410.

4. R. Rombach, A. Blattmann, D. Lorenz, P. Esser, B. Ommer, "High-Resolution Image Synthesis With Latent Diffusion Models" (2022; https://openaccess.thecvf.com/content/CVPR2022/html/Rombach_High-Resolution_Image_Synthesis_With_Latent_Diffusion_Models_CVPR_2022_paper.html), pp. 10684–10695.

5. J. Yu, X. Li, J. Y. Koh, H. Zhang, R. Pang, J. Qin, A. Ku, Y. Xu, J. Baldridge, Y. Wu, Vector-quantized Image Modeling with Improved VQGAN. arXiv arXiv:2110.04627 [Preprint] (2022). https://doi.org/10.48550/arXiv.2110.04627.

6. W. A. Eaton, V. Muñoz, S. J. Hagen, G. S. Jas, L. J. Lapidus, E. R. Henry, J. Hofrichter, Fast Kinetics and Mechanisms in Protein Folding1. *Annual Review of Biophysics* **29**, 327–359 (2000).

7. D. K. Ghosh, A. Ranjan, The metastable states of proteins. *Protein Science* **29**, 1559–1568 (2020).

8. H. Taketomi, Y. Ueda, N. Gō, Studies on protein folding, unfolding and fluctuations by computer simulation. I. The effect of specific amino acid sequence represented by specific inter-unit interactions. *Int J Pept Protein Res* **7**, 445–459 (1975).

9. J. Zhang, X. Lin, W. E, Y. Q. Gao, Machine-Learned Invertible Coarse Graining for Multiscale Molecular Modeling. arXiv arXiv:2305.01243 [Preprint] (2023). https://doi.org/10.48550/arXiv.2305.01243.

10. D. P. Kingma, M. Welling, Auto-Encoding Variational Bayes. arXiv arXiv:1312.6114 [Preprint] (2022). https://doi.org/10.48550/arXiv.1312.6114.

11. I. Goodfellow, J. Pouget-Abadie, M. Mirza, B. Xu, D. Warde-Farley, S. Ozair, A. Courville, Y. Bengio, "Generative Adversarial Nets" in *Advances in Neural Information Processing Systems* (Curran Associates, Inc., 2014; https://proceedings.neurips.cc/paper_files/paper/2014/hash/5ca3e9b122f61f8f06494c97b1afccf3-Abstract.html)vol. 27.

12. Y. Bengio, N. Léonard, A. Courville, Estimating or Propagating Gradients Through Stochastic Neurons for Conditional Computation. arXiv arXiv:1308.3432 [Preprint] (2013). https://doi.org/10.48550/arXiv.1308.3432.

13. L. Liu, C. Dong, X. Liu, B. Yu, J. Gao, Bridging Discrete and Backpropagation: Straight-Through and Beyond. *Advances in Neural Information Processing Systems* **36**, 12291–12311 (2023).

14. A. van den Oord, O. Vinyals, K. Kavukcuoglu, Neural Discrete Representation Learning. arXiv arXiv:1711.00937 [Preprint] (2018). https://doi.org/10.48550/arXiv.1711.00937.

15. P. Esser, R. Rombach, B. Ommer, "Taming Transformers for High-Resolution Image Synthesis" (2021; https://openaccess.thecvf.com/content/CVPR2021/html/Esser_Taming_Transformers_for_High-Resolution_Image_Synthesis_CVPR_2021_paper.html?ref=), pp. 12873–12883.

16. P. Sun, Y. Jiang, S. Chen, S. Zhang, B. Peng, P. Luo, Z. Yuan, Autoregressive Model Beats Diffusion: Llama for Scalable Image Generation. arXiv arXiv:2406.06525 [Preprint] (2024). https://doi.org/10.48550/arXiv.2406.06525.

17. K. Tian, Y. Jiang, Z. Yuan, B. Peng, L. Wang, Visual Autoregressive Modeling: Scalable Image Generation via Next-Scale Prediction. arXiv arXiv:2404.02905 [Preprint] (2024). https://doi.org/10.48550/arXiv.2404.02905.

18. M. Misiura, R. Shroff, R. Thyer, A. B. Kolomeisky, DLPacker: Deep learning for prediction of amino acid side chain conformations in proteins. *Proteins: Structure, Function, and Bioinformatics* **90**, 1278–1290 (2022).

19. J. Jumper, R. Evans, A. Pritzel, T. Green, M. Figurnov, O. Ronneberger, K. Tunyasuvunakool, R. Bates, A. Žídek, A. Potapenko, A. Bridgland, C. Meyer, S. A. A. Kohl, A. J. Ballard, A. Cowie, B. Romera-Paredes, S. Nikolov, R. Jain, J. Adler, T. Back, S. Petersen, D. Reiman, E. Clancy, M. Zielinski, M. Steinegger, M. Pacholska, T. Berghammer, S. Bodenstein, D. Silver, O. Vinyals, A. W. Senior, K. Kavukcuoglu, P. Kohli, D. Hassabis, Highly accurate protein structure prediction with AlphaFold. *Nature* **596**, 583–589 (2021).

20. J. Zhang, Y.-K. Lei, Y. Zhou, Y. I. Yang, Y. Q. Gao, Molecular CT: Unifying Geometry and Representation Learning for Molecules at Different Scales. arXiv arXiv:2012.11816 [Preprint] (2023). https://doi.org/10.48550/arXiv.2012.11816.

21. L. McInnes, J. Healy, J. Melville, UMAP: Uniform Manifold Approximation and Projection for Dimension Reduction. arXiv arXiv:1802.03426 [Preprint] (2020). https://doi.org/10.48550/arXiv.1802.03426.

22. X. Chen, Y. Duan, R. Houthooft, J. Schulman, I. Sutskever, P. Abbeel, InfoGAN: Interpretable Representation Learning by Information Maximizing Generative Adversarial Nets. arXiv arXiv:1606.03657 [Preprint] (2016). https://doi.org/10.48550/arXiv.1606.03657.

23. I. J. Goodfellow, J. Shlens, C. Szegedy, Explaining and Harnessing Adversarial Examples. arXiv arXiv:1412.6572 [Preprint] (2015). https://doi.org/10.48550/arXiv.1412.6572.

24. M. Mirza, S. Osindero, Conditional Generative Adversarial Nets. arXiv arXiv:1411.1784 [Preprint] (2014). https://doi.org/10.48550/arXiv.1411.1784.

25. H. K. Wayment-Steele, A. Ojoawo, R. Otten, J. M. Apitz, W. Pitsawong, M. Hömberger, S. Ovchinnikov, L. Colwell, D. Kern, Predicting multiple conformations via sequence clustering and AlphaFold2. *Nature* **625**, 832–839 (2024).

26. C.-L. Li, W.-C. Chang, Y. Cheng, Y. Yang, B. Póczos, MMD GAN: Towards Deeper Understanding of Moment Matching Network. arXiv arXiv:1705.08584 [Preprint] (2017). https://doi.org/10.48550/arXiv.1705.08584.

27. B. Jing, B. Berger, T. Jaakkola, AlphaFold Meets Flow Matching for Generating Protein Ensembles. arXiv arXiv:2402.04845 [Preprint] (2024). https://doi.org/10.48550/arXiv.2402.04845.

28. A. Łańcucki, J. Chorowski, G. Sanchez, R. Marxer, N. Chen, H. J. G. A. Dolfing, S. Khurana, T. Alumäe, A. Laurent, Robust Training of Vector Quantized Bottleneck Models. arXiv arXiv:2005.08520 [Preprint] (2020). https://doi.org/10.48550/arXiv.2005.08520.

29. H. Chang, H. Zhang, L. Jiang, C. Liu, W. T. Freeman, MaskGIT: Masked Generative Image Transformer. arXiv arXiv:2202.04200 [Preprint] (2022). https://doi.org/10.48550/arXiv.2202.04200.

30. J. Zhang, Y.-K. Lei, X. Che, Z. Zhang, Y. I. Yang, Y. Q. Gao, Deep Representation Learning for Complex Free-Energy Landscapes. *The Journal of Physical Chemistry Letters*, doi: 10.1021/acs.jpclett.9b02012 (2019).

31. X. Lin, Y. Xia, Y. Huang, S. Liu, J. Zhang, Y. Q. Gao, Versatile Molecular Editing via Multimodal and Group-optimized Generative Learning. ChemRxiv [Preprint] (2024). https://doi.org/10.26434/chemrxiv-2023-j2n6l-v2.

32. S. Liu, J. Zhang, H. Chu, M. Wang, B. Xue, N. Ni, J. Yu, Y. Xie, Z. Chen, M. Chen, Y. Liu, P. Patra, F. Xu, J. Chen, Z. Wang, L. Yang, F. Yu, L. Chen, Y. Q. Gao, PSP: Million-level Protein Sequence Dataset for Protein Structure Prediction. arXiv arXiv:2206.12240 [Preprint] (2022). https://doi.org/10.48550/arXiv.2206.12240.

33. Clustering predicted structures at the scale of the known protein universe | Nature. https://www.nature.com/articles/s41586-023-06510-w.

34. Multi-domain and complex protein structure prediction using inter-domain interactions from deep learning | Communications Biology. https://www.nature.com/articles/s42003-023-05610-7.

35. M. Gao, D. Nakajima An, J. M. Parks, J. Skolnick, AF2Complex predicts direct physical interactions in multimeric proteins with deep learning. *Nat Commun* **13**, 1744 (2022).

36. T. Hrabe, Z. Li, M. Sedova, P. Rotkiewicz, L. Jaroszewski, A. Godzik, PDBFlex: exploring flexibility in protein structures. *Nucleic Acids Res* **44**, D423-428 (2016).

37. R. Aggarwal, A. Gupta, U. D. Priyakumar, APObind: A Dataset of Ligand Unbound Protein Conformations for Machine Learning Applications in De Novo Drug Design. arXiv arXiv:2108.09926 [Preprint] (2021). https://doi.org/10.48550/arXiv.2108.09926.

38. Antibody structure prediction using interpretable deep learning. *Patterns* **3**, 100406 (2022).

39. M. Varadi, D. Bertoni, P. Magana, U. Paramval, I. Pidruchna, M. Radhakrishnan, M. Tsenkov, S. Nair, M. Mirdita, J. Yeo, O. Kovalevskiy, K. Tunyasuvunakool, A. Laydon, A. Žídek, H. Tomlinson, D. Hariharan, J. Abrahamson, T. Green, J. Jumper, E. Birney, M. Steinegger, D. Hassabis, S. Velankar, AlphaFold Protein Structure Database in 2024: providing structure coverage for over 214 million protein sequences. *Nucleic Acids Research* **52**, D368–D375 (2024).

40. M. van Kempen, S. S. Kim, C. Tumescheit, M. Mirdita, J. Lee, C. L. M. Gilchrist, J. Söding, M. Steinegger, Fast and accurate protein structure search with Foldseek. *Nat Biotechnol* **42**, 243–246 (2024).

41. S. Basu, B. Wallner, DockQ: A Quality Measure for Protein-Protein Docking Models. *PLOS ONE* **11**, e0161879 (2016).

42. D. del Alamo, D. Sala, H. S. Mchaourab, J. Meiler, Sampling alternative conformational states of transporters and receptors with AlphaFold2. *eLife* **11**, e75751 (2022).

43. J. Zhang, S. Liu, M. Chen, H. Chu, M. Wang, Z. Wang, J. Yu, N. Ni, F. Yu, D. Chen, Y. I. Yang, B. Xue, L. Yang, Y. Liu, Y. Q. Gao, Unsupervisedly Prompting AlphaFold2 for Accurate Few-Shot Protein Structure Prediction. *Journal of Chemical Theory and Computation*, doi: 10.1021/acs.jctc.3c00528 (2023).

44. Y. Kalakoti, B. Wallner, AFsample2: Predicting multiple conformations and ensembles with AlphaFold2. bioRxiv [Preprint] (2024). https://doi.org/10.1101/2024.05.28.596195.

45. Z. Sun, Q. Liu, G. Qu, Y. Feng, M. T. Reetz, Utility of B-Factors in Protein Science: Interpreting Rigidity, Flexibility, and Internal Motion and Engineering Thermostability. *Chem. Rev.* **119**, 1626–1665 (2019).

46. P. Ma, D.-W. Li, R. Brüschweiler, Predicting protein flexibility with AlphaFold. *Proteins: Structure, Function, and Bioinformatics* **91**, 847–855 (2023).

47. openmm/pdbfixer, OpenMM (2024); https://github.com/openmm/pdbfixer.

48. P. Ayaz, A. Lyczek, Y. Paung, V. R. Mingione, R. E. Iacob, P. W. de Waal, J. R. Engen, M. A. Seeliger, Y. Shan, D. E. Shaw, Structural mechanism of a drug-binding process involving a large conformational change of the protein target. *Nat Commun* **14**, 1885 (2023).

49. M. T. Degiacomi, Coupling Molecular Dynamics and Deep Learning to Mine Protein Conformational Space. *Structure* **27**, 1034-1040.e3 (2019).

50. R. Šink, M. Kotnik, A. Zega, H. Barreteau, S. Gobec, D. Blanot, A. Dessen, C. Contreras-Martel, Crystallographic Study of Peptidoglycan Biosynthesis Enzyme MurD: Domain Movement Revisited. *PLOS ONE* **11**, e0152075 (2016).

51. J. Zhao, M. Elgeti, E. S. O'Brien, C. P. Sár, A. EI Daibani, J. Heng, X. Sun, E. White, T. Che, W. L. Hubbell, B. K. Kobilka, C. Chen, Ligand efficacy modulates conformational dynamics of the µ-opioid receptor. *Nature* **629**, 474–480 (2024).

52. H. Ding, Y. Xu, Q. Chen, H. Dai, Y. Tang, J. Wu, Y. Shi, Solution Structure of Human SUMO-3 C47S and Its Binding Surface for Ubc9,. *Biochemistry* **44**, 2790–2799 (2005).

53. G. Cornilescu, J. L. Marquardt, M. Ottiger, A. Bax, Validation of Protein Structure from Anisotropic Carbonyl Chemical Shifts in a Dilute Liquid Crystalline Phase. *J. Am. Chem. Soc.* **120**, 6836–6837 (1998).

54. C. Genis, K. H. Sippel, N. Case, W. Cao, B. S. Avvaru, L. J. Tartaglia, L. Govindasamy, C. Tu, M. Agbandje-McKenna, D. N. Silverman, C. J. Rosser, R. McKenna, Design of a Carbonic Anhydrase IX Active-Site Mimic To Screen Inhibitors for Possible Anticancer Properties. *Biochemistry* **48**, 1322–1331 (2009).

55. S. Bouyain, D. J. Watkins, The protein tyrosine phosphatases PTPRZ and PTPRG bind to distinct members of the contactin family of neural recognition molecules. *Proceedings of the National Academy of Sciences* **107**, 2443–2448 (2010).
